# The characterisation of shellac resin by flow injection and liquid chromatography coupled with electrospray ionisation and mass spectrometry

**DOI:** 10.1038/s41598-017-14907-7

**Published:** 2017-11-01

**Authors:** Diego Tamburini, Joanne Dyer, Ilaria Bonaduce

**Affiliations:** 1 Department of Scientific Research, The British Museum, Great Russell Street, WC1B 3DG, London, United Kingdom; 2 Department of Chemistry and Industrial Chemistry, University of Pisa, via G. Moruzzi 13, I-56124 Pisa, Italy

## Abstract

A strategy based on electrospray ionisation (ESI) in negative mode coupled with quadrupole-time of flight (Q-ToF) detection techniques was adopted to characterise some samples of shellac resin. Flow injection analysis (FIA) was used to investigate the distribution of the components of the resin. Eight groups of compounds with increasing masses were detected and assigned to free acids, esters and polyesters with up to eight units. High pressure liquid chromatography (HPLC) enabled the compounds to be chromatographically separated. Accurate molecular masses and tandem mass (MS/MS) spectra interpretation were used to characterise the different compounds, assigning and/or suggesting molecular structures. In some cases, highly detailed information about the ester linkages was provided by the MS/MS spectra, enabling the different isomers to be distinguished. Oxidation products were also identified in the samples and differences were observed in terms of hydrolysis and oxidation. In addition to providing the first characterisation of shellac by HPLC-ESI-Q-ToF and an atlas of MS/MS spectra of shellac components, this work demonstrates the suitability of the proposed strategy for characterising the resin, and provides the identification of previously unknown degradation products and minor components. This represents a significant step forward in the chemical knowledge of this material.

## Introduction

Shellac is a natural resin secreted by the Indian scale insect *Kerria lacca*, also known as *Laccifer lacca* Kerr. The insect infests branches of various trees, such as *Butea monosperma* (Lam.) Taub. (synonym *Butea frondosa* Rosch)*, Vachellia nilotica* (L.) P.J.H.Hurter & Mabb. (formerly classified as *Acacia arabica* Willd) and *Ficus religiosa* Linn, commonly found in India, Thailand, Myanmar and south China^1^. At the end of the 16^th^ century, shellac was introduced into Europe^2^, and has been widely used since as an adhesive, sealing, insulating and coating material for several applications, including the production of musical instruments, the protection of vinyl records, and the insulation of radios and electrical tools^3–5^. Currently, shellac is still used as a wood-finishing material and a coating for pharmaceuticals and food products^6^. Shellac has also been used in the field of conservation, as a varnish for wooden objects (“French polish”) and mural paintings, or as an adhesive for ceramics^7^.

Shellac has a complex chemical composition, which may vary slightly depending on the host tree, species of the insect and environmental conditions. It is a mixture of resin (70–80%), wax (6–7%) and colourant molecules (4–8%)^8^, obtained by refining sticklac, which is the material collected directly from the plant. After washing the sticklac, most of the water-soluble material (e.g. laccaic acids) is removed and seedlac is obtained. If the seedlac then solely undergoes the traditional melting filtration process, the product obtained is termed wax-containing shellac or common shellac. Further refinements can be performed in order to remove colour, by bleaching (bleached shellac), or to remove the waxy components by solvent extraction (dewaxed shellac)^9^.

The resin is composed of two major fractions commonly referred to as “soft” (30%) and “hard” - or “pure” – (70%) resin^10^. These fractions are complex mixtures of different mono- and polyesters of hydroxyaliphatic acids, *i.e.;* 9,10,16-trihydroxyhexadecanoic (aleuritic acid) and 6-hydroxytetradecanoic acids (butolic acid), and sesquiterpenoid acids, *i.e.;* jalaric and laccijalaric acids. The relative amounts of these acids and the manner in which the corresponding esters are formed differ for the “soft” and “hard” resin, with the “soft” resin generally having a lower molecular weight and the “hard” resin constituting the backbone of the material^8,10–15^. Minor compounds are also present among the hydroxyaliphatic acids, such as 6-oxotetradecanoic acid, 16-hydroxyhexadecanoic acid, 6-hydroxyhexadec-9-enoic acid, 9,10-dihydroxytetradecanoic acid and 9,10-dihydroxyhexadecanoic acid^16^. 8-hydroxyacids have also been detected as shellac components^6^. With regards to the terpenoid acids, the disproportionation products of jalaric and laccijalaric acids are usually detected^8,10,17,18^. In these compounds the original aldehyde group is substituted by a hydroxyl or carboxyl group. The reduction of jalaric acid produces laksholic acid and the oxidation produces shellolic acid. Laccilaksholic and laccishellolic acids are the corresponding products of the reduction and oxidation of laccijalaric acid. The species are present in both epimeric forms^10,17^. These molecules are produced *via* a Cannizzaro-type reaction, which occurs in alkaline conditions, *i.e*. during the saponification step usually needed to cleave the ester bonds for subsequent analysis of the resin by GC-MS^8^. However, shellolic and laccishellolic acids are also original components of the resin^17^, produced to a lesser extent by the natural oxidation of aldehyde moieties with ageing.

In terms of chemical analysis, FT-IR^19^, Raman^20^ and fluorescence^3^ spectroscopies have been successfully applied for the identification of shellac. However, these techniques fail in the specific identification of aged natural resins or in the presence of complex mixtures^21^. GC-MS and Py-GC-MS have enabled detailed information to be obtained on the constituting acids of the resin at the molecular level^6,17,18,22,23^. Phenomena taking place with ageing, including crosslinking, inter-molecular esterification and formation of unsaturated compounds, were also highlighted^18,19^. Due to the macromolecular nature of shellac, gas chromatography requires chemical or thermal pre-treatment, in order to obtain components suitable for gas chromatographic analysis. As a result, information on the macromolecular composition of the resin is inevitably lost.

HPLC-MS is a powerful state-of-the-art technique suitable for the analysis of large and polar molecules, which can be obtained from the sample after minimal pre-treatment. It is used routinely for the analysis of many materials in samples collected from works of art^24,25^. Preliminary investigations on shellac by HPLC-MS have been performed previously^18,26^. In these cases the resin was analysed after solvent extraction, which enabled the preservation of important and informative bonds between molecules, thus allowing a few ester structures to be accessed and investigated. However, the potential of the technique to provide a more thorough characterisation of the resin was not explored.

In this work, this methodology is taken a step further, for the first time, to provide a complete characterisation of a reference sample of shellac by FIA-ESI-Q-ToF and HPLC-ESI-QToF, enabling the composition of this material to be investigated at the molecular level, and both ester structures and the nature of some previously unidentified degradation products to be clarified.

To verify the applicability of this approach to aged shellac samples and for a preliminary exploration of the feasibility of this methodology to probe the nature of the changes in molecular structure and composition as a result of the ageing process, two historic shellac samples from the natural history collection of the Salvemini Collection in Florence were also investigated and the results compared with the reference material.

## Results and Discussion

### Reference sample – sample S0 from the British Museum collection

#### FIA-ESI-Q-ToF

**Figure 1:**
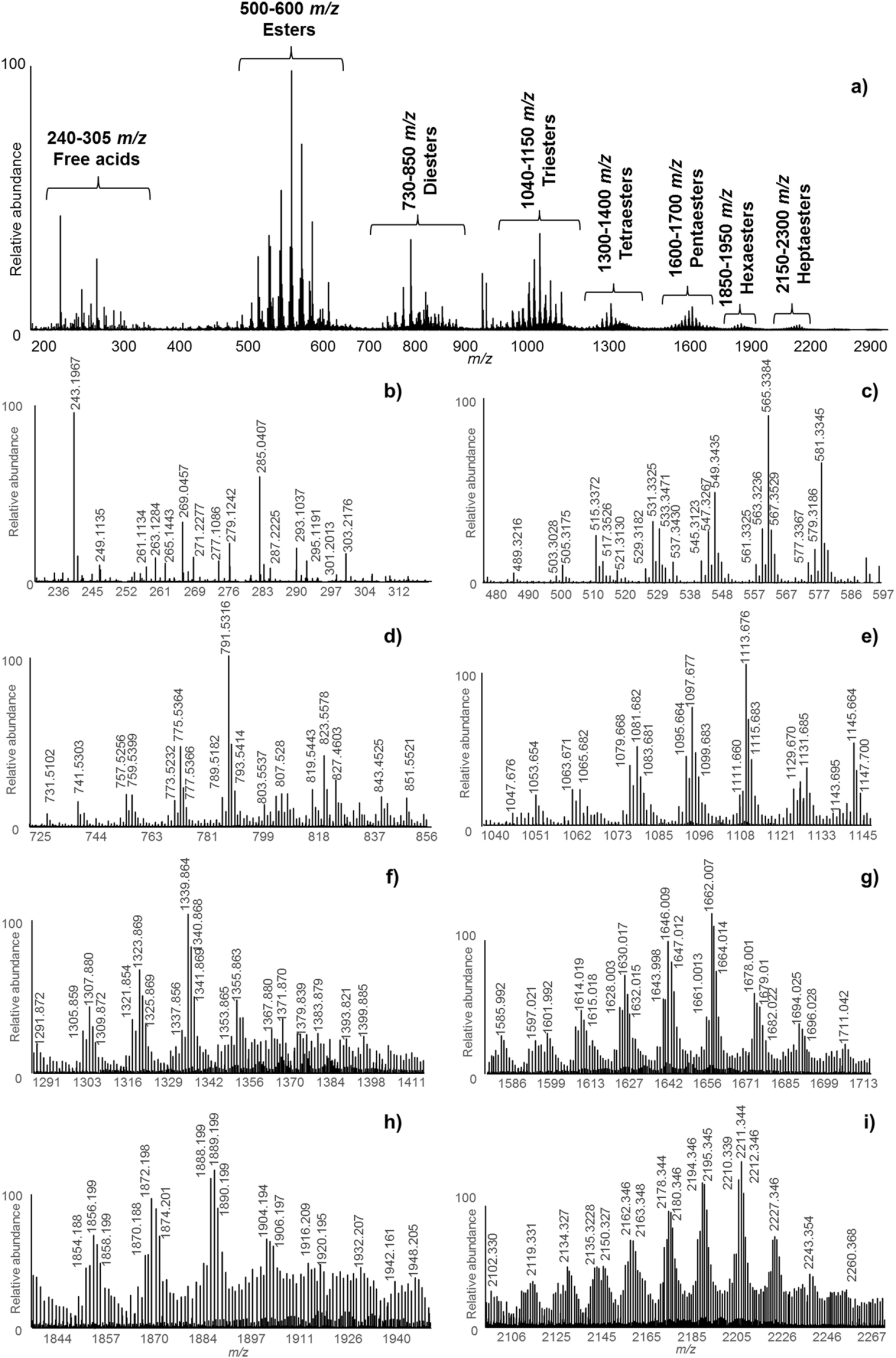
Overall mass spectrum (**a**) obtained by FIA-ESI-Q-ToF of the methanol extracts of samples S0 (*m/z* range 200–3000). Detailed areas, corresponding to the clusters of (**b**) free acids, (**c**) esters, (**d**) diesters, (**e**) triesters, (**f**) tetraesters, (**g**) pentaesters, (**h**) hexaesters, and (**i**) heptaesters.

Figure 1a shows the overall mass spectrum obtained by FIA analysis of sample S0. The spectrum showed eight main *m/z* clusters, *i.e.;* ~240–305 *m/z*, 500–600 *m/z*, 730–850 *m/z*, 1040–1150 *m/z*, 1300–1400 *m/z*, 1600–1700 *m/z*, 1850–1950 *m/z*, 2150–2300 *m/z*. Assignments for some of the main *m/z* peaks observed in the FIA-ESI mass spectrum are reported in Table 1. These assignments were suggested based on high resolution mass measurements, which enabled the chemical formulas to be identified. The mass differences expressed in ppm - diff(ppm) - between experimental and calculated mass values are also reported in Table 1. The instrumental accuracy in mass measurements resulted in a diff(ppm) always below 2 ppm, with the exception of a few cases where it resulted between 2 and 3 ppm. Considering that 2 ppm of 500 u corresponds to 0.001 u, the uncertainty of the measurement is related to the third decimal digit for molecules whose molecular weight is above 500 u. On the other hand, for molecules whose molecular weight is below 500 u, the uncertainty of the measurement is related to the fourth decimal digit. This means, from a theoretical point of view, that the fourth decimal digit can be mathematically exploited for the identification of molecules whose molecular weight is below 500 u, whereas the third decimal digit should be considered for molecules whose molecular weight is above 500 u. However, as the diff(ppm) in our results was in most cases much lower than 2 ppm, the fourth decimal digit was considered for molecules up to *ca*. 900 u weight. Therefore, in this article *m/z* values below 900 are presented with four decimal digits, whereas *m/z* values above 900 are presented with three decimal digits. It has to be underlined that high resolution mass measurements are generally not sufficient for the identification of a molecule, as one chemical formula can correspond to several isomers. Table 1 contains more data than the simple assignments of FIA-ESI-Q-ToF data to chemical formulas. These represent the summary of all findings of the paper, which are fully discussed in the following paragraphs.

**Table 1:** List of compounds identified in the shellac samples.

***Label***	***Name***	***RT (min)***	***Experimental mass [M-H]*** ^***−***^	***Calculated mass [M-H]*** ^***−***^	***Diff (ppm)***	***Chemical formula***	***MSMS ESI (-) fragment ions***
**Free acids**							
6-oxo-C14	6-oxotetradecanoic acid	8.43	241.1811	241.1809	−0.75	C_14_H_26_O_3_	223, 197, 157, 139
But	Butolic acid	8.13	243.1967	243.1966	−0.54	C_14_H_28_O_3_	197, 141, 85
9,10-diOH-C14	9,10-dihydroxytetradecanoic acid	7.13	259.1919	259.1915	−1.60	C_14_H_28_O_4_	241, 233, 203, 183, 171, 155, 143, 127, 115
Ox-Ljal	Oxidised laccijalaric acid	5.86	261.1134	261.1132	−0.64	C_15_H_18_O_4_	217, 189, 149, 121
Ox-Ljal	Oxidised laccijalaric acid	6.46	261.1134	261.1132	−0.64	C_15_H_18_O_4_	217, 189, 149, 121
Ljal	Laccijalaric acid	5.45	263.1284	263.1289	1.83	C_15_H_20_O_4_	219, 201, 189, 165, 137, 107
Llak	Laccilaksholic acid	5.00	265.1443	265.1445	0.87	C_15_H_22_O_4_	221, 193, 175, 139, 109
E-Llak	Epilaccilaksholic acid	5.42	265.1443	265.1445	0.87	C_15_H_22_O_4_	221, 193, 175, 139, 109
16OH-C16:1	16-hydroxyhexadec-9-enoic acid	8.08	269.2120	269.2122	0.81	C_16_H_30_O_3_	251, 223, 155, 141, 127, 113
16OH-C16	16-hydroxyhexadecanoic acid	8.89	271.2277	271.2279	0.62	C_16_H_32_O_3_	253, 225, 155, 141, 127, 113
Ox-Jal	Oxidised jalaric acid	5.38	277.1086	277.1081	−1.63	C_15_H_18_O_5_	233, 215, 189, 171, 135, 107
Jal	Jalaric acid	4.05	279.1242	279.1238	−1.44	C_15_H_20_O_5_	261, 235, 217, 189, 147, 121, 107
Lshel	Laccishellolic acid	4.52	279.1242	279.1238	−1.44	C_15_H_20_O_5_	235, 217, 187, 103
Jal	Jalaric acid isomer	4.99	279.1242	279.1238	−1.44	C_15_H_20_O_5_	261, 235, 217, 189, 147, 121, 107
Lak	Laksholic	5.35	281.1399	281.1394	−1.60	C_15_H_22_O_5_	263, 237, 219, 201, 147
9,10-diOH-C16:2	9,10-dihydroxyhexadecadienoic acid	6.48	283.1920	283.1915	−1.82	C_16_H_28_O_4_	239, 143, 141, 117
9,10-diOH-C16:1	9,10-dihydroxyhexadecenoic acid	6.26	285.2075	285.2071	−1.28	C_16_H_30_O_4_	239, 171, 143, 125
9,10-diOH-C16	9,10-dihydroxyhexadecanoic acid	6.51	287.2225	287.2228	0.98	C_16_H_32_O_4_	269, 251, 211, 171, 143, 127, 113
Ox-Shel	Oxidised shellolic acid	4.53	293.1037	293.1031	−2.17	C_15_H_18_O_6_	249, 221, 205, 189, 117
Ox-Eshel	Oxidised epishellolic acid	5.08	293.1037	293.1031	−2.17	C_15_H_18_O_6_	249, 221, 205, 189, 117
Shel	Shellolic acid	2.17	295.1191	295.1187	−1.31	C_15_H_20_O_6_	251, 249, 207, 177, 147, 121
E-shel	Epishellolic acid	2.52	295.1191	295.1187	−1.31	C_15_H_20_O_6_	251, 249, 207, 177, 147, 121
Ox-Al	Oxidised aleuritic acid	5.79	301.2013	301.2020	2.47	C_16_H_30_O_5_	283, 265, 171, 127
Al	Aleuritic acid	5.55	303.2176	303.2177	0.32	C_16_H_32_O_5_	285, 267, 227, 201, 171, 155, 127, 113
**Esters**							
Ljal-But	Laccijalaric-butolic	9.79	489.3216	489.3222	1.15	C_29_H_46_O_6_	243, 225
Ljal-(9,10-diOH-C14:1)	Laccijalaric-(9,10-dihydroxytetradecenoic)	8.79	503.3028	503.3014	−2.72	C_29_H_44_O_7_	257, 217
Ljal-(9,10-diOH-C14)	Laccijalaric-(9,10-dihydroxytetradecanoic)	8.58	505.3175	505.3171	−0.83	C_29_H_46_O_7_	259
Ljal-(16OH-C16:1)	Laccijalaric-(16-hydroxyhexadec-9-enoic)	9.98	515.3372	515.3378	1.19	C_31_H_48_O_6_	269
Ljal-(16OH-C16)	Laccijalaric-(16-hydroxyhexadecanoic)	10.44	517.3526	517.3535	1.66	C_31_H_50_O_6_	271, 247
Jal-(9,10-diOH-C14)	Jalaric-(9,10-dihydroxytetradecanoic)	7.95	521.3130	521.3120	−1.93	C_29_H_46_O_8_	279, 261, 217
Ljal-(9,10-diOH-C16:2)	Laccijalaric-(9,10-dihydroxyhexadecadienoic)	8.37	529.3182	529.3171	−2.12	C_31_H_46_O_7_	283
Ljal-(9,10-diOH-C16:1)	Laccijalaric-(9,10-dihydroxyhexadecenoic)	8.22	531.3325	531.3327	0.43	C_31_H_48_O_7_	285
Jal-(16OH-C16:1)	Jalaric-(16-hydroxyhexadec-9-enoic)	8.97	531.3325	531.3327	0.43	C_31_H_48_O_7_	269, 261, 217, 161, 121
Ljal-(9,10-diOH-C16)	Laccijalaric-(9,10-dihydroxyhexadecanoic)	8.47	533.3471	533.3484	2.39	C_31_H_50_O_7_	287
Jal-(16OH-C16)	Jalaric-(16-hydroxyhexadecanoic)	9.40	533.3471	533.3484	2.39	C_31_H_50_O_7_	287, 271, 261, 217, 189, 149, 121
Al-?	Unidentified ester I	7.06	535.3267	535.3276	1.76	C_30_H_48_O_8_	303, 277, 247, 233, 185, 133
Al-?	Unidentified ester II	6.89	537.3430	537.3433	0.54	C_30_H_50_O_8_	303, 287, 249
Ljal-(Ox-Al)	Laccijalaric- oxidised aleuritic	7.82	547.3267	547.3276	1.72	C_31_H_48_O_8_	503, 301, 261, 217, 147
Ljal-Al	Laccijalaric-aleuritic	7.50	549.3435	549.3433	−0.38	C_31_H_50_O_8_	303, 287, 261, 217, 161, 121
Al-Llak	Aleuritic-Llak	6.40	551.3220	551.3226	1.01	C_30_H_48_O_9_	303, 265, 247, 185
Al-?	Unidentified ester III	6.07	553.3386	553.3382	−0.71	C_30_H_50_O_9_	303, 285, 249, 149
Jal-(Ox-Al)	jalaric- oxidised aleuritic	7.11	563.3236	563.3226	−1.85	C_31_H_48_O_9_	301, 277, 261, 233, 217, 147
Jal-Al	Jalaric-aleuritic	6.66	565.3384	565.3382	−0.34	C_31_H_50_O_9_	303, 261, 217, 121
Jal-Al	Jalaric-aleuritic	6.78	565.3384	565.3382	−0.34	C_31_H_50_O_9_	303, 285, 261, 217, 121
Lshel-Al	Laccishellolic-aleuritic	7.02	565.3384	565.3382	−0.34	C_31_H_50_O_9_	303, 279, 261, 233, 217, 147
Lak-Al	Laksholic-aleuritic	7.52	567.3529	567.3539	1.68	C_31_H_52_O_9_	303, 287, 279, 263, 217
Elak-Al	Epilaksholic-aleuritic	6.55	567.3529	567.3539	1.68	C_31_H_52_O_9_	303, 287, 279, 263, 217
Shel-(ox-Al)	Shellolic-aleuritic oxidised	6.93	579.3186	579.3175	−1.94	C_31_H_48_O_10_	301, 277, 261, 233, 217, 121
Shel-Al	Shellolic-aleuritic	6.26	581.3345	581.3331	−2.37	C_31_H_50_O_10_	303, 277, 233
Diesters**							
Ljal-(9,10-diOH-C14)-But	Laccijalaric-(9,10-dihydroxytetradecanoic)-butolic	11.48	731.5102	731.5104	0.21	C_43_H_72_O_9_	485, 259, 243
Ljal-(16OH-C16:1)-But	Laccijalaric-(16-hydroxyhexadec-9-enoic)-butolic	12.91	741.5303	741.5311	1.07	C_45_H_74_O_8_	495, 269, 243
Ljal-(9,10-diOH-C16:2)-But	Laccijalaric-(9,10-dihydroxyhexadecadienoic)-butolic	11.66	755.5100	755.5104	0.47	C_45_H_72_O_9_	509, 283, 243
Jal-(16OH-C16:1)-But	Jalaric-(16-hydroxyhexadec-9-enoic)-butolic	12.31	757.5256	757.5260	0.54	C_45_H_74_O_9_	495, 269, 261, 243, 217
Ljal-(9,10-diOH-C16)-But-	Laccijalaric-(9,10-dihydroxyhexadecanoic)-butolic	11.75	759.5399	759.5417	2.31	C_45_H_76_O_9_	513, 287, 243
Jal-(16OH-C16)-But	Jalaric-(16-hydroxyhexadecanoic)-butolic	12.72	759.5399	759.5417	2.31	C_45_H_76_O_9_	513, 279, 261, 243, 227
Ljal-Al-But	Laccijalaric-aleuritic-butolic	10.44	775.5364	775.5366	0.22	C_45_H_76_O_10_	529, 303, 285, 243
Ljal-Al-But	Laccijalaric-aleuritic-butolic	10.95	775.5364	775.5366	0.22	C_45_H_76_O_10_	529, 303, 261, 243, 217
Jal-(9,10-diOH-C16)-But-	Jalaric-(9,10-dihydroxyhexadecanoic)-butolic	11.10	775.5364	775.5366	0.22	C_45_H_76_O_10_	513, 287, 261, 243, 217
Jal-Al-But	Jalaric-aleuritic-butolic	9.68	791.5316	791.5315	−0.14	C_45_H_76_O_11_	529, 303, 285, 261, 243, 217
Jal-Al-But	Jalaric-aleuritic-butolic	10.19	791.5316	791.5315	−0.14	C_45_H_76_O_11_	529, 303, 261, 243, 217
Shel-Al-But	Shellolic-aleuritic-butolic	9.11	807.5280	807.5264	−1.98	C_45_H_76_O_12_	529, 303, 285, 277, 243, 233, 217
Shel-Al-But	Shellolic-aleuritic-butolic	9.69	807.5280	807.5264	−1.98	C_45_H_76_O_12_	529, 277, 243
Ljal-Al-Jal	Laccijalaric-aleuritic-jalaric	8.29	811.4646	811.4638	−0.98	C_46_H_68_O_12_	565, 549, 531, 303, 279, 261, 217
Jal-Al-Jal-	Jalaric-aleuritic-jalaric	7.31	827.4603	827.4587	−1.91	C_46_H_68_O_13_	565, 279, 261, 217
Jal-Shel-Al	Jalaric-aleuritic-shellolic-	6.94	843.4525	843.4536	1.34	C_46_H_68_O_14_	581, 565, 303, 277, 233
Jal-Shel-Al	Jalaric-aleuritic-shellolic	7.32	843.4525	843.4536	1.34	C_46_H_68_O_14_	581, 565, 547, 303, 293, 277, 261, 233
Jal-Al-Al	Jalaric-aleuritic-aleuritic	7.53	851.5521	851.5526	0.61	C_47_H_80_O_16_	589, 563, 303, 261
Triesters**							
Ljal-Al-(16OH-C16:1)-Ljal	Laccijalaric-aleuritic-(16-hydroxyhexadec-9-enoic)-laccijalaric	11.54	1047.676	1047.678	1.54	C_62_H_96_O_13_	801, 549, 531, 515, 303, 269, 263
Ljal-(9,10-diOH-C16)-Ljal-(16OH-C16:1)	Laccijalaric-(16-hydroxyhexadec-9-enoic)-laccijalaric-(9,10-dihydroxyhexadecanoic)	11.92	1047.676	1047.678	1.54	C_62_H_96_O_13_	801, 777, 555, 531, 517, 503, 487, 287, 269, 263
Ljal-Al-Jal-(16OH-C16:1)	Laccijalaric-aleuritic-jalaric-(16-hydroxyhexadec-9-enoic)	11.10	1063.671	1063.673	1.44	C_62_H_96_O_14_	817, 793, 573, 55, 531, 487, 303, 285, 269, 261, 217
Jal-Al-(16OH-C16:1)-Jal-	Jalaric-aleuritic-(16-hydroxyhexadec-9-enoic)-jalaric-	9.89	1079.667	1079.668	1.06	C_62_H_96_O_15_	817, 547, 531, 303, 269, 261, 217
Ljal-Al-Jal-(9,10-diOH-C16:1)	Laccijalaric-aleuritic-(9,10-dihydroxyhexadecenoic)-jalaric	10.11	1079.667	1079.668	1.06	C_62_H_96_O_15_	833, 817, 571, 547, 531, 503, 303, 285, 277, 261, 217
Ljal-Al-Al-Ljal	Laccijalaric-aleuritic-aleuritic-laccijalaric	9.49	1081.682	1081.683	1.29	C_62_H_98_O_15_	835, 589, 549, 303, 263
Jal-Al-(9,10-diOH-C16:1)-Jal-	Jalaric-aleuritic-(9,10-dihydroxyhexadecenoic)-jalaric-	9.56	1095.664	1095.663	−1.59	C_62_H_96_O_16_	833, 587, 571, 547, 303, 285, 277, 261, 217
Jal-Al-(16OH-C16:1)-Shel	Jalaric-aleuritic-(16-hydroxyhexadec-9-enoic)-shellolic	9.27	1095.664	1095.663	−1.59	C_62_H_96_O_16_	833, 571, 547, 529, 303, 277, 269, 261, 233, 217
Ljal-Al-Al-Jal	Laccijalaric-aleuritic-aleuritic-jalaric	8.78	1097.677	1097.678	1.01	C_62_H_98_O_16_	851, 835, 589, 565, 549, 303, 287, 261, 217
Ljal-Al-Al-Jal	Laccijalaric-aleuritic-aleuritic-jalaric	9.03	1097.677	1097.678	1.01	C_62_H_98_O_16_	851, 835, 589, 565, 547, 303, 261, 217
Jal-Al-Al-Jal	Jalaric-aleuritic-aleuritic-jalaric	8.17	1113.676	1113.673	−2.22	C_62_H_98_O_17_	851, 589, 565, 547, 303, 261
Jal-Al-Al-Shel	Jalaric-aleuritic-aleuritic-shellolic	7.68	1129.670	1129.668	−1.82	C_62_H_98_O_18_	867, 851, 589, 565, 303, 277, 261, 233
Shel-Al-Al-Shel	Shellolic-aleuritic-aleuritic-shellolic	7.26	1145.664	1145.663	−0.65	C_62_H_98_O_19_	867, 605, 581, 563, 303, 277, 233
Tetraesters**							
Ljal-Al-But-Al-Ljal	Laccijalaric-aleuritic-butolic-aleuritic-laccijalaric	12.12	1307.880	1307.876	2.18	C_76_H_124_O_17_	1061, 815, 775, 571, 549, 529, 303, 263, 261, 243
Ljal-Al-But-Al-Jal	Laccijalaric-aleuritic-butolic-aleuritic-jalaric	11.77	1323.869	1323.872	1.65	C_76_H_124_O_18_	1077, 1061, 815, 775, 547, 529, 513, 303, 261, 243
Jal-Al-But-Al-Jal	Jalaric-aleuritic-butolic-aleuritic-jalaric	10.95	1339.864	1339.866	1.50	C_76_H_124_O_19_	1077, 815, 791, 773, 565, 547, 529, 303, 261, 243
Ljal-Al-But-Al-Shel	Laccijalaric-aleuritic-butolic-aleuritic-shel	11.31	1339.864	1339.866	1.50	C_76_H_124_O_19_	1077, 815, 773, 547, 529, 303, 277, 261, 243
Ljal-Al-But-Al-Jal	Jalaric-aleuritic-butolic-aleuritic-shel	10.25	1355.863	1355.861	−1.46	C_76_H_124_O_20_	1093, 1077, 815, 791, 589, 563, 547, 529, 303, 277, 261, 233
Ljal-Al-Jal-Al-Jal	Laccijalaric-aleuritic-jalaric-aleuritic-jalaric	9.20	1359.800	1359.799	−0.79	C_77_H_116_O_20_	1113, 1097, 851, 835, 565, 547, 303, 279, 261
Jal-Al-Jal-Al-Jal	Jalaric-aleuritic-jalaric-aleuritic-jalaric	8.56	1375.796	1375.794	−1.36	C_77_H_116_O_21_	1113, 851, 827, 565, 547, 303, 279, 261, 217
Jal-Al-Jal-Al-Shel	Jalaric-aleuritic-jalaric-aleuritic-shellolic	8.21	1391.790	1391.789	−1.11	C_77_H_116_O_22_	1129, 1113, 851, 809, 581, 565, 547, 303, 277, 261, 233
Jal-Al-Al-Al-Jal	Jalaric-aleuritic-aleuritic-aleuritic-jalaric	8.65	1399.885	1399.888	2.09	C_78_H_128_O_21_	1137, 875, 851, 589, 565, 547, 303, 261, 217
Pentaesters** ^**a**^							
Ljal-Ljal-Ljal-Al-Al-Al	Laccijalaric-laccijalaric-laccijalaric-aleuritic-aleuritic-aleuritic	11.22	1614.019	1614.023	2.41	C_93_H_146_O_22_	1368, 1353, 1121, 1105, 875, 835, 589, 547, 531, 303, 261
Ljal-Ljal-Jal-Al-Al-Al	Laccijalaric-laccijalaric-jalaric-aleuritic-aleuritic-aleuritic	10.30	1630.017	1630.018	0.50	C_93_H_146_O_23_	1383, 1369, 1137, 1121, 1098, 851, 835, 589, 565, 549, 531, 303, 261
Ljal-Ljal-Shel-Al-Al-(9,10-diOH-C16)	Laccijalaric-laccijalaric-shellolic-aleuritic-aleuritic-(9,10-dihydroxyhexadecanoic)	11.40	1630.017	1630.018	0.50	C_93_H_146_O_23_	1383, 1367, 1121, 1105, 1081, 835, 819, 589, 573, 565, 547, 531, 303, 287, 277, 261
Ljal-Jal-Jal-Al-Al-Al	Laccijalaric-jalaric-jalaric-aleuritic-aleuritic-aleuritic	9.62	1646.010	1646.013	2.02	C_93_H_146_O_24_	1400, 1384, 1138, 1122, 1096, 1081, 852, 833, 589, 565, 547, 303, 261
Ljal-Jal-Shel-Al-Al-(9,10-diOH-C16)	Laccijalaric-jalaric-shellolic-aleuritic-aleuritic-(9,10-dihydroxyhexadecanoic)	10.46	1646.010	1646.013	2.02	C_93_H_146_O_24_	1384, 1122, 1096, 1079, 876, 851, 835, 589, 573, 565, 547, 531, 303, 287, 277, 261
Jal-Jal-Jal-Al-Al-Al	Jalaric-jalaric-jalaric-aleuritic-aleuritic-aleuritic	9.08	1662.007	1662.008	0.87	C_93_H_146_O_25_	1400, 1138, 1114, 1096, 852, 810, 589, 565, 547, 303, 261
Ljal-Jal-Shel-Al-Al-Al	Laccijalaric-jalaric-shellolic-aleuritic-aleuritic-aleuritic	9.42	1662.007	1662.008	0.87	C_93_H_146_O_25_	1400, 1358, 1138, 1096, 851, 834, 809, 589, 565, 547, 303, 277, 261
Jal-Jal-Shel-Al-Al-Al	Jalaric-jalaric-shellolic-aleuritic-aleuritic-aleuritic	9.07	1678.001	1678.003	1.11	C_93_H_146_O_26_	1416, 1400, 1138, 1113, 1096, 852, 589, 565, 547, 303, 277, 261, 233
Hexaesters** ^**a**^							
Jal-Jal-Jal-Al-Al-Al-But	Jalaric-jalaric-jalaric-aleuritic-aleuritic-aleuritic-butolic	11.44	1888.199	1888.201	1.23	C_107_H_172_O_27_	1627, 1364, 1348, 1113, 1077, 851, 792, 589, 565, 547, 529, 303, 261, 243
Heptaesters** ^**a**^							
Ljal-Jal-Jal-Jal-Al-Al-Al-Al	Laccijalaric-jalaric-jalaric-jalaric-aleuritic-aleuritic-aleuritic-aleuritic	10.52	2195.346	2194.348	1.16	C_124_H_194_O_32_	1933, 1687, 1671, 1400, 1358, 1138, 1097, 1080, 851, 810, 781, 565, 547, 303, 261
Jal-Jal-Jal-Jal-Al-Al-Al-Al	Jalaric-jalaric-jalaric-jalaric-aleuritic-aleuritic-aleuritic-aleuritic	9.82	2210.339	2210.343	1.61	C_124_H_194_O_33_	1949, 1687, 1401, 1358, 1138, 1113, 1096, 852, 833, 589, 565, 547, 303, 261
Jal-Jal-Jal-Shel-Al-Al-Al-Al	Jalaric-jalaric-jalaric-shellolic-aleuritic-aleuritic-aleuritic-aleuritic	8.45	2226.341	2226.338	−1.18	C_124_H_194_O_34_	1965, 1948, 1687, 1374, 1113, 851, 565, 303, 261

Retention times – RT –, difference between the experimental and calculated masses of the deprotonated molecules [M-H]^−^ – diff(ppm) –, chemical formulas and nominal masses* of the fragment ions present in the MS/MS spectra are reported. *The measured accurate masses of the fragment ions are reported in the corresponding MS/MS spectra shown in the Appendix (Supplementary Information). The accuracy of measurements in MS/MS experiments is usually lower than MS experiments, but four decimal digits have been displayed for consistency.

**Isomers are generally present with differences in the relative abundances of *m/z* peaks. The retention time of the most abundant isomer is reported.

^**a**^The exact order in which the acids are linked in the polyester was not ascertained.

Free acids, esters and polyesters composed of up to eight units were detected, in agreement with the shellac composition reported in the literature^15^. Details of the clusters identified by FIA for sample S0, showing the experimental mass values, are shown in Fig. 1b–i. The colourants erythrolaccin ([M]^−^ = 285.0407* m/z*) and deoxyerythrolaccin ([M]^−^ = 269.0457* m/z*) were also detected and identified by comparison with the data present in the in-house database of dye molecules at the British Museum.

The relative abundance of the clusters observed should not be taken as indicative of the actual quantitative composition of the resin, as the ionisation yields of different compounds is likely to differ. In addition, as the size of the molecules increases, the possibility of multiply charged ions being formed in the ESI source also increases. Doubly charged ions are in fact visible in the mass spectra of the triesters, tetraesters, pentaesters, hexaesters and heptaesters (Fig. 1e–i), with minor relative abundances with respect to singly charged ions.

#### HPLC-ESI-Q-ToF

Methanol extracts of all the samples analysed produced very complex Total Ion Current (TIC) chromatograms (Figure S1 of the Supplementary Information). HPLC-ESI-Q-ToF analyses enabled the MS/MS spectra to be acquired. Although the ultimate identification of a molecule is achieved by the comparison of its retention time, accurate mass and MS/MS spectrum with that of a standard molecule, this was not possible in our case, as standard molecules of most shellac components are not commercially available. However, aleuritic acid is commercially available and it was analysed. The comparison of the retention time, the accurate mass and the MS/MS spectrum confirmed our interpretation and is shown in Figure S2 of the Supplementary Information. For the other molecules, the study of MS/MS fragmentation patterns^27,28^, together with high resolution mass measurements, helped recognition of the main shellac free sesquiterpenoid and aliphatic acids^6,22^. Generally, the interpretation of the MS/MS fragmentation was also crucial in elucidating the molecular structure of the esters, sometimes allowing differentiation between isomers. Molecules never reported in the literature were also detected, and again, MS data interpretation was used to hypothesise structures. The distinction between epimers, *e.g*. shellolic and epishellolic acids, was only tentative. For such molecules, two compounds were present with very similar MS/MS spectra and we assumed that these were the two epimeric forms.

**Figure 2:**
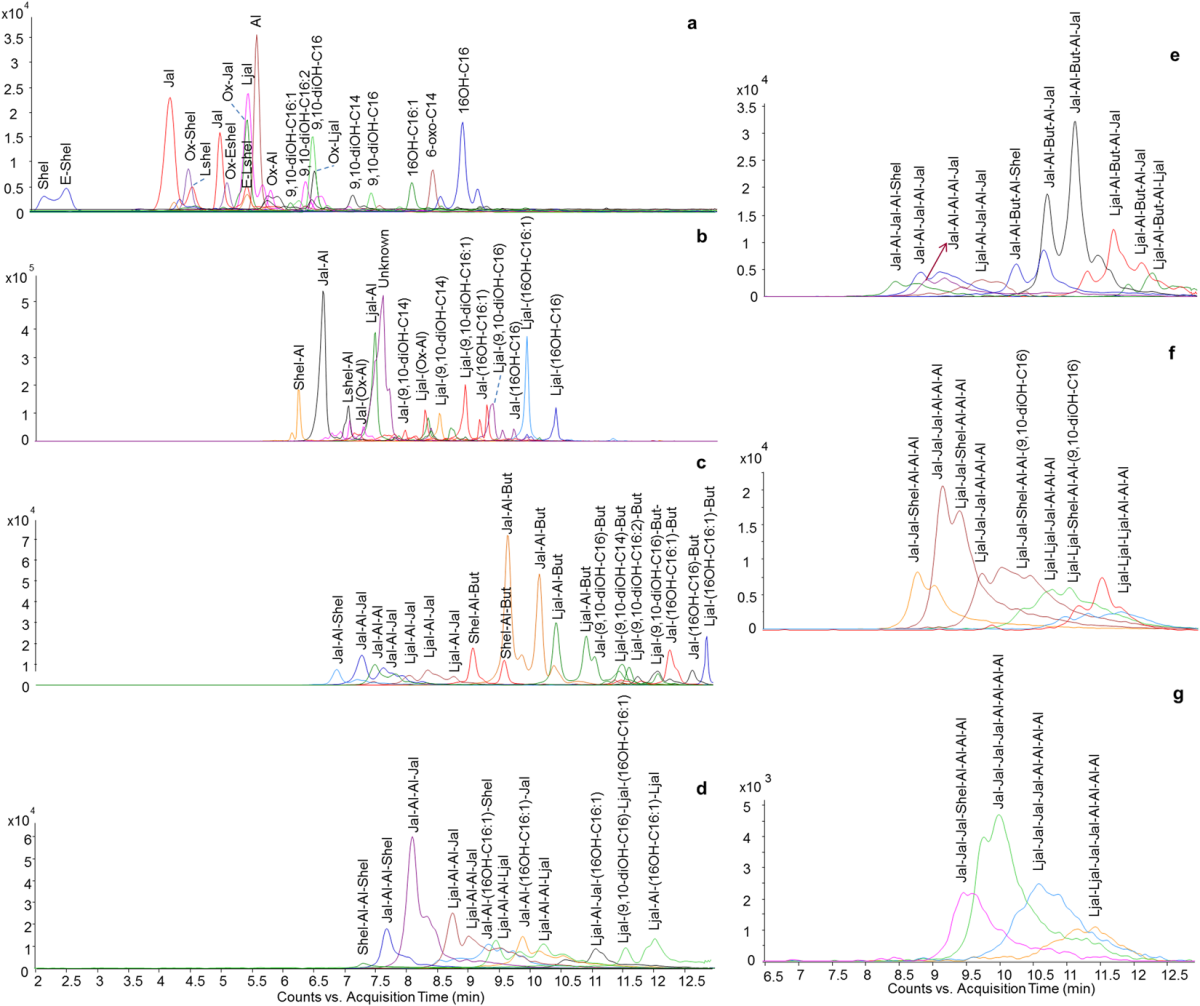
Extracted ion chromatograms (EICs) obtained by HPLC-ESI-Q-ToF of sample S0. (**a**) free acids, (**b**) esters, (**c**) diesters, (**d**) triesters, (**e**) tetraesters, (**f**) pentaesters and hexaesters, (**g**) heptaesters. Labels refer to Table 1. The EIC of butolic acid is not reported, as its abundance was one order of magnitude higher than the other free acids.

Figure 2 reports the Extracted Ion Chromatograms (EICs) for sample S0, showing the presence of chromatographic peaks ascribable to free acids, esters, diesters, triesters, tetraesters, pentaesters, hexaesters and heptaesters. Table 1 lists peak attribution, together with selected details of the fragmentation observed in the MS/MS spectra acquired with negative ionisation. The MS/MS spectra are reported in the Appendix (Supplementary Information).

Among the free acids, butolic acid showed the chromatographic peak with the highest relative abundance, as a consequence of its ionisation yield, and the fact that this acid is considered to be poorly included in the backbone of shellac, instead functioning as a plasticiser^8^. Peaks ascribable to esters dominated the chromatogram. Jalaric-aleuritic and laccijalaric-aleuritic esters gave the most abundant peaks among the monoesters, in agreement with the literature reporting that these three acids are the main components of shellac^15^. Significant relative abundances were also shown by the esters of 9,10-dihydroxytetradecanoic acid and 16-hydroxyhexadecanoic acid with sesquiterpenoid acids. These hydroxyacids have been reported to be present as minor components of shellac analysed following saponification^16^. As the number of units increases in the polyesters, an increase in the number of isomers was observed. This resulted in a general broadening of the peaks for high masses, as the isomers were not completely chromatographically resolved. For this reason, the retention times used to indicate the compounds in Table 1 refer to the most relatively abundant isomer. Only in the case of clearly different MS/MS spectra acquired, more than one isomer is reported. The distinction between isomers in terms of bond positions was sometimes impossible, but the order by which the constituting units were linked was determined in most cases up to the tetraesters. MS data interpretation is discussed in detail in the following paragraphs.

**Figure 3:**
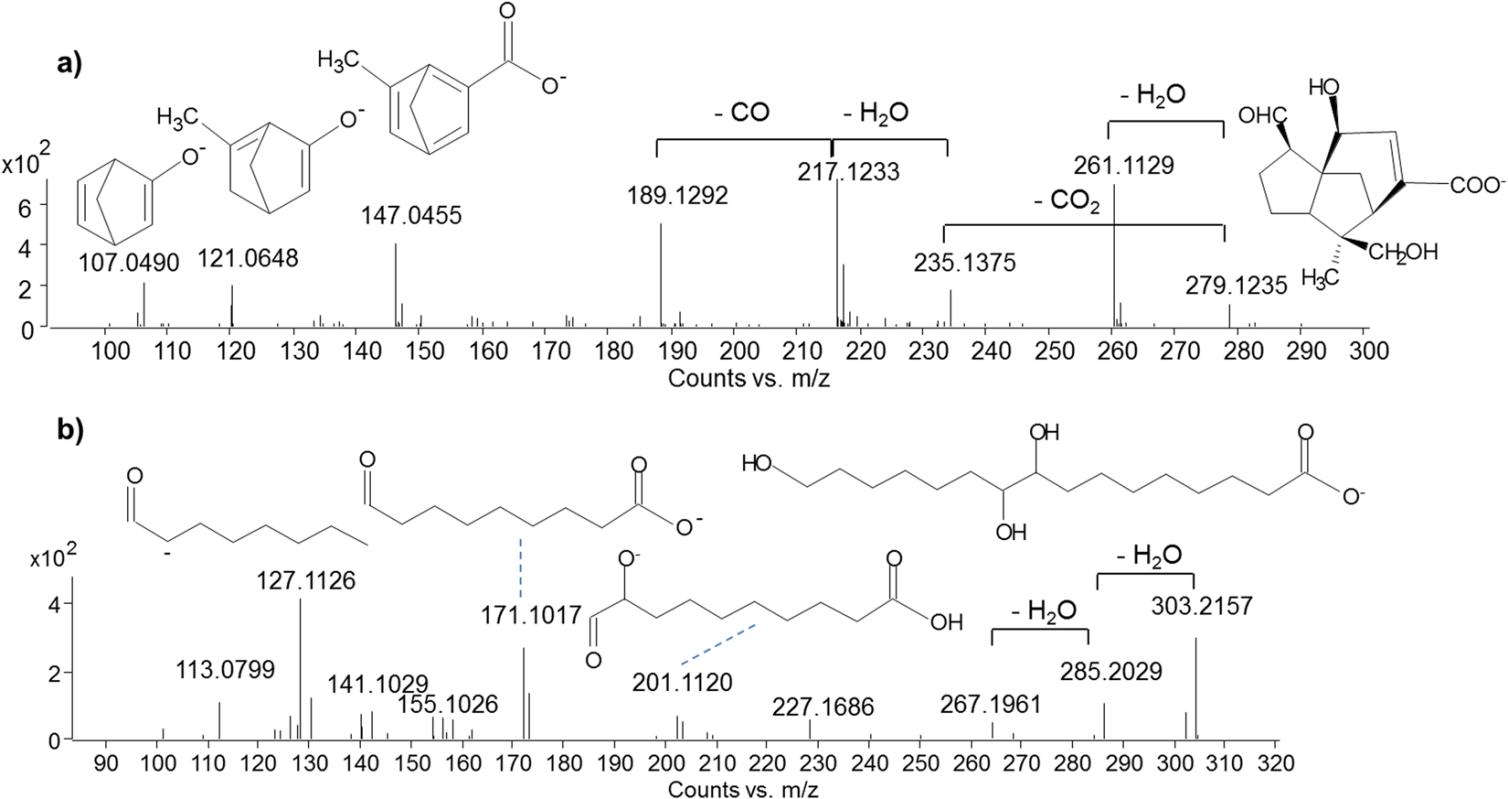
MS/MS spectra obtained in ESI (−) mode of (**a**) jalaric acid and (**b**) aleuritic acid.

*Sesquiterpenoid and hydroxyaliphatic acids*. The fragmentation pattern of sesquiterpenoid acids showed the typical losses of 43.9898 u (CO_2_), 30.0106 u (CH_2_O), 27.9949 u (CO) and 18.0106 u (H_2_O)^29^, derived from the loss of their functional groups, as shown in Fig. 3a for jalaric acid. These masses are calculated masses. These do not always correspond to the experimental masses reported in the figures. However, the difference between the two masses is below 2 ppm in most cases. Lower *m/z* values corresponded to further cleavage of sesquiterpenoid structures. Some tentative structures for the fragment ions are shown in Fig. 3a.

The MS/MS spectra obtained for aliphatic hydroxyacids, such as butolic acid, aleuritic acid and their derivatives also showed neutral losses of H_2_O and CO_2_ molecules. In addition, fragment ions produced by the cleavage at locations corresponding to the hydroxyl positions were present in the MS/MS spectra (Fig. 3b)^27^. As an example, the fragment ion with *m/z* 171.1027 (C_9_H_15_O_3_^−^) derives from the cleavage between C_9_-C_10_ (Fig. 3b), thus indicating the presence of a hydroxyl group at the C_9_ position. This fragment ion is present in all the 9,10-dihydroxycarboxylic acids (Table 1, Appendix).

**Figure 4:**
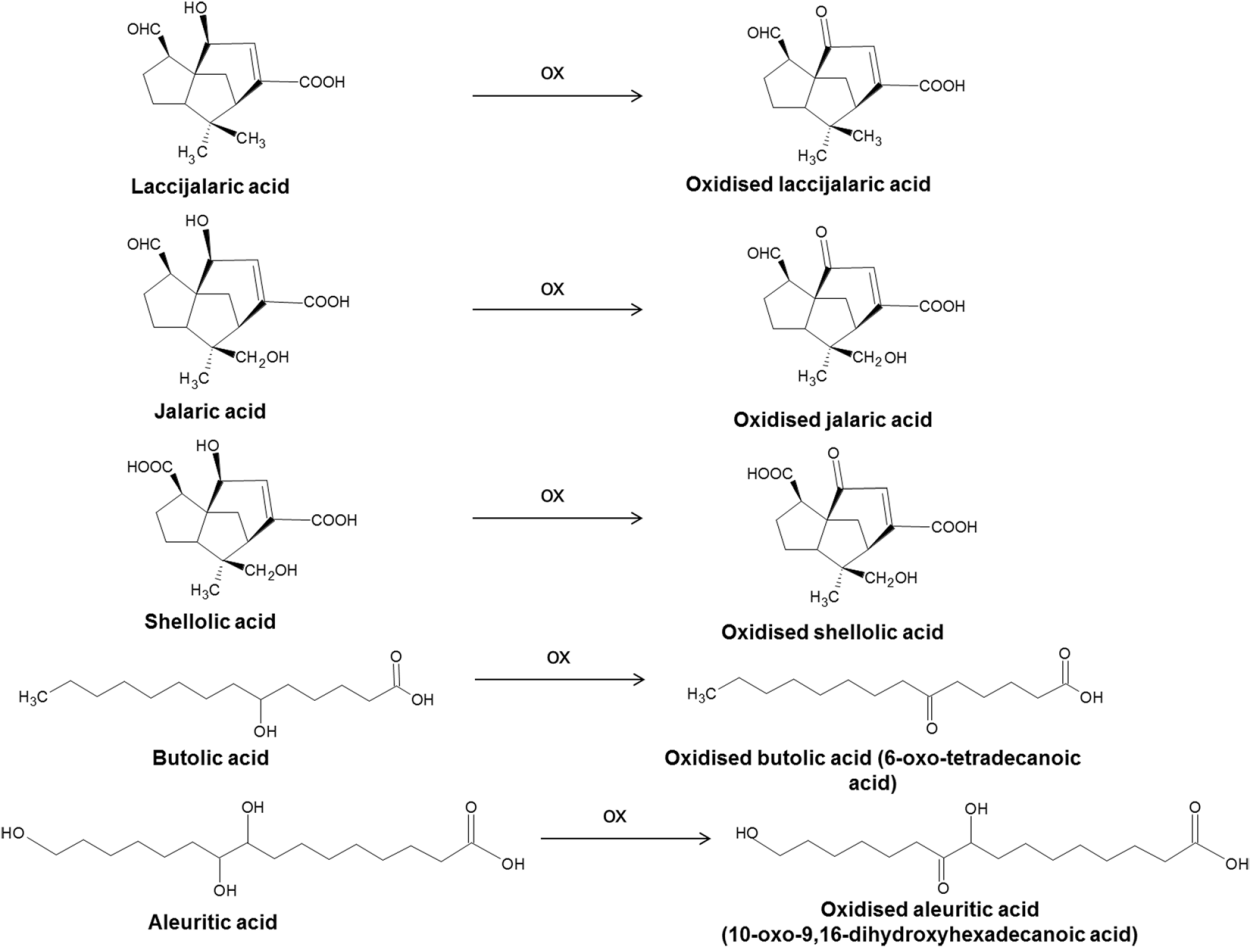
Shellac oxidised compounds detected in the samples analysed.

Among the free acids, a series of molecules was detected, whose masses did not correspond to any of the shellac components reported in the literature. All these compounds showed a 2 u (-H_2_) mass difference compared to the main shellac components (laccijalaric, jalaric, shellolic, butolic, aleuritic acids). The interpretation of the MS/MS spectra of these compounds led to the hypothesis that the hydroxyl group of the terpenoid acids had undergone an oxidation reaction, leading to the formation of a keto group, as shown in Fig. 4. For jalaric acid and shellolic acids, which contain two hydroxyl groups, the oxidised position was most likely the one on the ring, as the formation of a keto group at the indicated position results in a stable bond conjugated with the double bond on the sesquiterpenoid ring. Similar oxidation reactions were also observed for butolic and aleuritic acids. The compounds produced by this reaction are referred to as oxidised acids in Table 1 and their mass spectra are reported in the Appendix. 6-oxotetradecanoic acid, an oxidation product of butolic acid, is the only one of these compounds already reported in the literature^16^. Similarly, an oxidised aleuritic acid was also detected. The MS/MS spectrum (Appendix) suggested that the oxidation could have occurred on the hydroxyl group at the C_10_ position. In fact, the fragment ion with *m/z* 171.1027, indicating the hydroxyl group at the C_9_ position, was present in the spectrum. On the contrary, the fragment ion with *m/z* 201.1120 (C_10_H_17_O_4_^−^), present in the MS/MS spectrum of aleuritic acid (Fig. 3b) and indicative of the hydroxyl group at the C_10_ position, was absent. Moreover, the presence of the fragment ion with *m/z* 99.0830 (C_6_H_11_O^−^) was most likely formed by the cleavage between C_10_ and C_11_. If a keto group was present at the C_16_ position, this fragment would have resulted in a C_6_H_9_O^−^ ion with *m/z* 97.0659.

**Figure 5:**
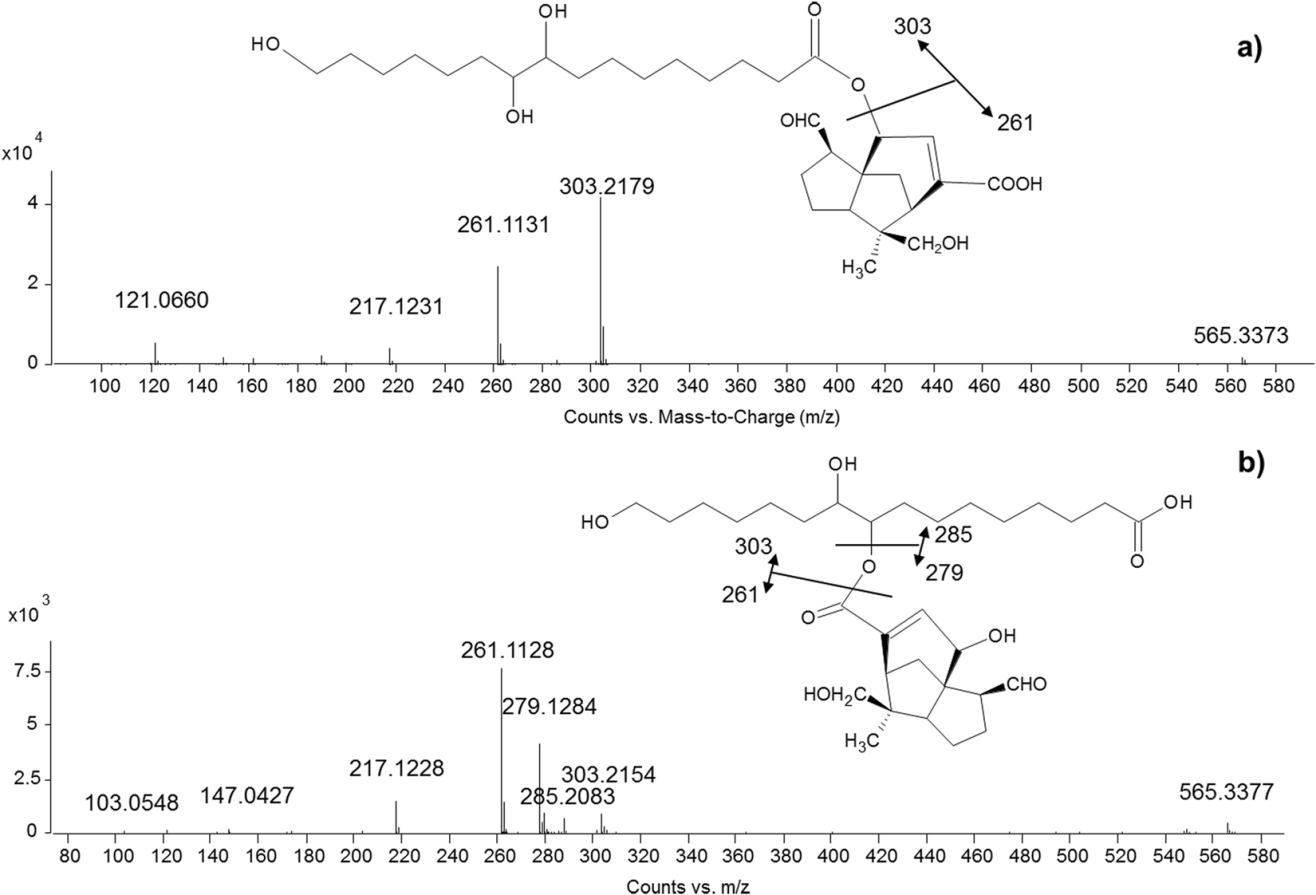
MS/MS spectra obtained in ESI (−) mode of (**a**) jalaric-aleuritic ester formed through the carboxylic group on the aleuritic acid and (**b**) jalaric-aleuritic ester formed through the carboxylic group on the jalaric acid.

*Esters*. The MS/MS spectra of the esters generally showed the deprotonated molecules and were dominated by the fragment ions produced by the cleavage of the ester bond. Fragment ions ascribable to the fragmentation of the sesquiterpenoid acids were also often present. Polyhydroxyaliphatic acids and sesquiterpenoid acids can form esters *via* different pathways. In the case of aleuritic acid, the most common ways are by reaction of the carboxylic group of the aleuritic acid with the hydroxyl group of the sesquiterpenoid acid, or reaction of the hydroxyl group at the C_9_ position of aleuritic acid with the carboxylic group of the sesquiterpenoid acid^13^. It has also been shown that the frequency of the former bond is twice more abundant than the latter one^13^. In our results, isomers were commonly found with both slightly different retention times and abundances of *m/z* peaks in the MS/MS spectra. In some cases, the interpretation of the MS/MS spectrum was straightforward, as one main fragmentation pathway was evident, as shown in Fig. 5a for the ester between jalaric and aleuritic acids. In other cases, the fragmentation was slightly more complex, and the MS/MS spectra showed additional *m/z* peaks, as shown in Fig. 5b for an isomer of the ester between jalaric and aleuritic acids. According to the relative abundance of the isomers and to the relative ability of the carbonyl unit to accommodate the negative charge, we believe that in the former case the carboxylic group of the aleuritic acid is involved in the ester bond, whereas in the latter case the carboxylic group of the sesquiterpenoid acid is involved in the ester bond.

**Figure 6:**
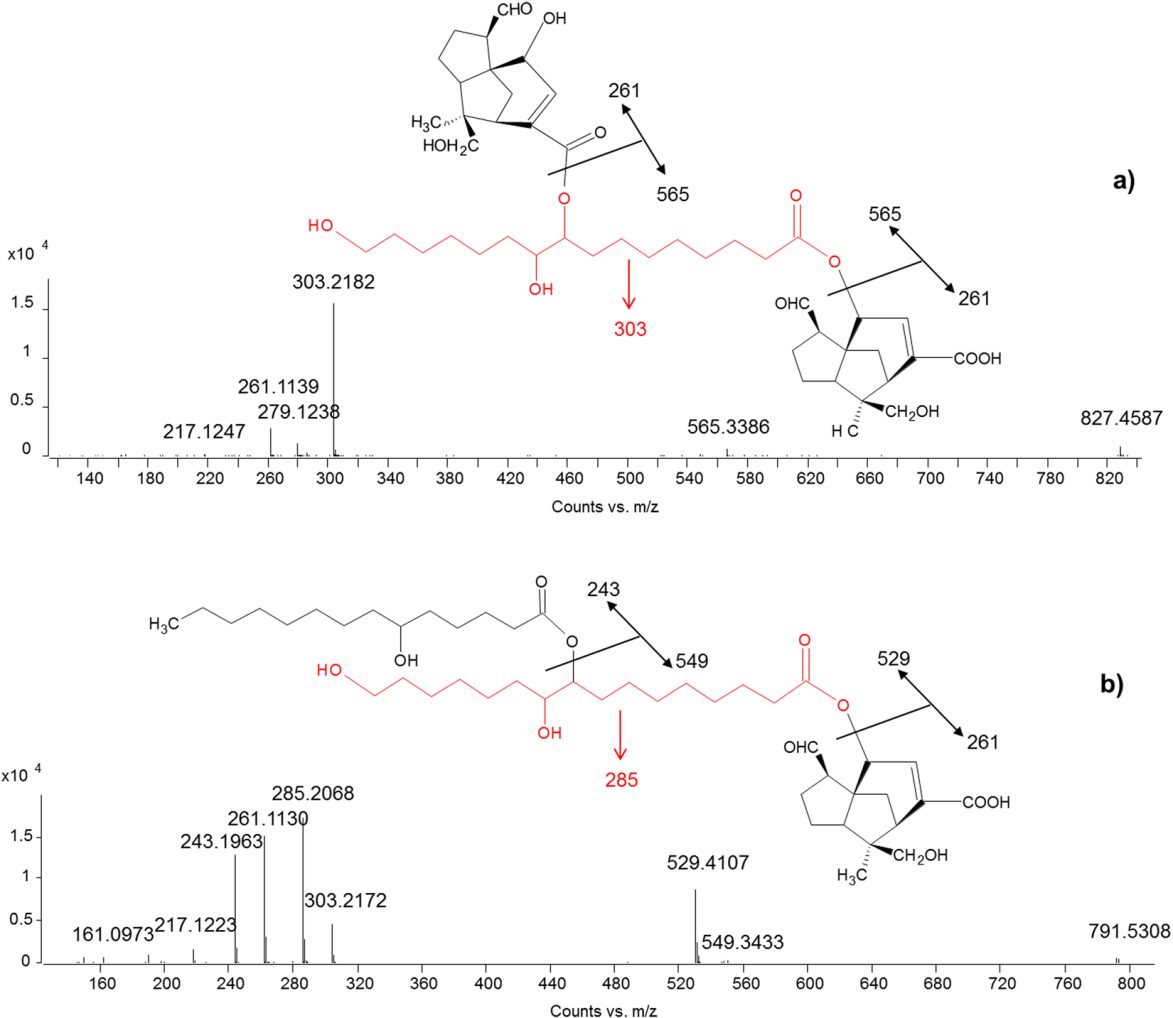
MS/MS spectra obtained in ESI (−) mode of (**a**) jalaric-aleuritic-jalaric diester (A-B-A configuration) and (**b**) jalaric-aleuritic-butolic diester (A-B-C configuration).

*Diesters*. Several isomers were generally detected for each diester and, in most cases, MS/MS spectra enabled the molecular structures to be established. In principle, we can expect several possibilities, including A-B-B, A-B-A, A-B-C, A-C-B, B-A-C, *etc*. configurations, where the letters indicate the different constituting acids. The fragment ion observed at the highest *m/z* values is indicative of the cleavage at the side position of the diester. In A-B-A configurations, the same molecule occupies the side position, thus resulting in a single fragmentation peak in this region of the spectrum. As an example, the spectrum of jalaric-aleuritic-jalaric diester, reported in Fig. 6a, shows a single peak at 565.3386 *m/z* (calculated mass 565.3382), resulting from the loss of a jalaric acid, and a peak at *m/z* 303.2182 (corresponding to the mass of aleuritic acid – calculated mass 303.2177), resulting from the loss of the other jalaric acid unit.

In A-B-B or A-B-C configurations, two different molecules occupy the side positions; therefore two fragment ions are expected. This is the case of the jalaric-aleuritic-butolic diester, which shows a *m/z* peak at 549.3433 (calculated mass 549.3433) deriving from the loss of butolic acid and a *m/z* peak at 529.4107 (calculate mass 529.4110) deriving from the loss of jalaric acid, as shown in Fig. 6b. Also in this case, the second fragmentation reveals the nature of the central molecule (aleuritic acid). In some other cases where butolic acid occupies the side position, the fragment ion deriving from its loss presented very low relative intensity. It has to be noted that aleuritic acid and its derivatives proved to always occupy the central position (see Appendix).

**Figure 7:**
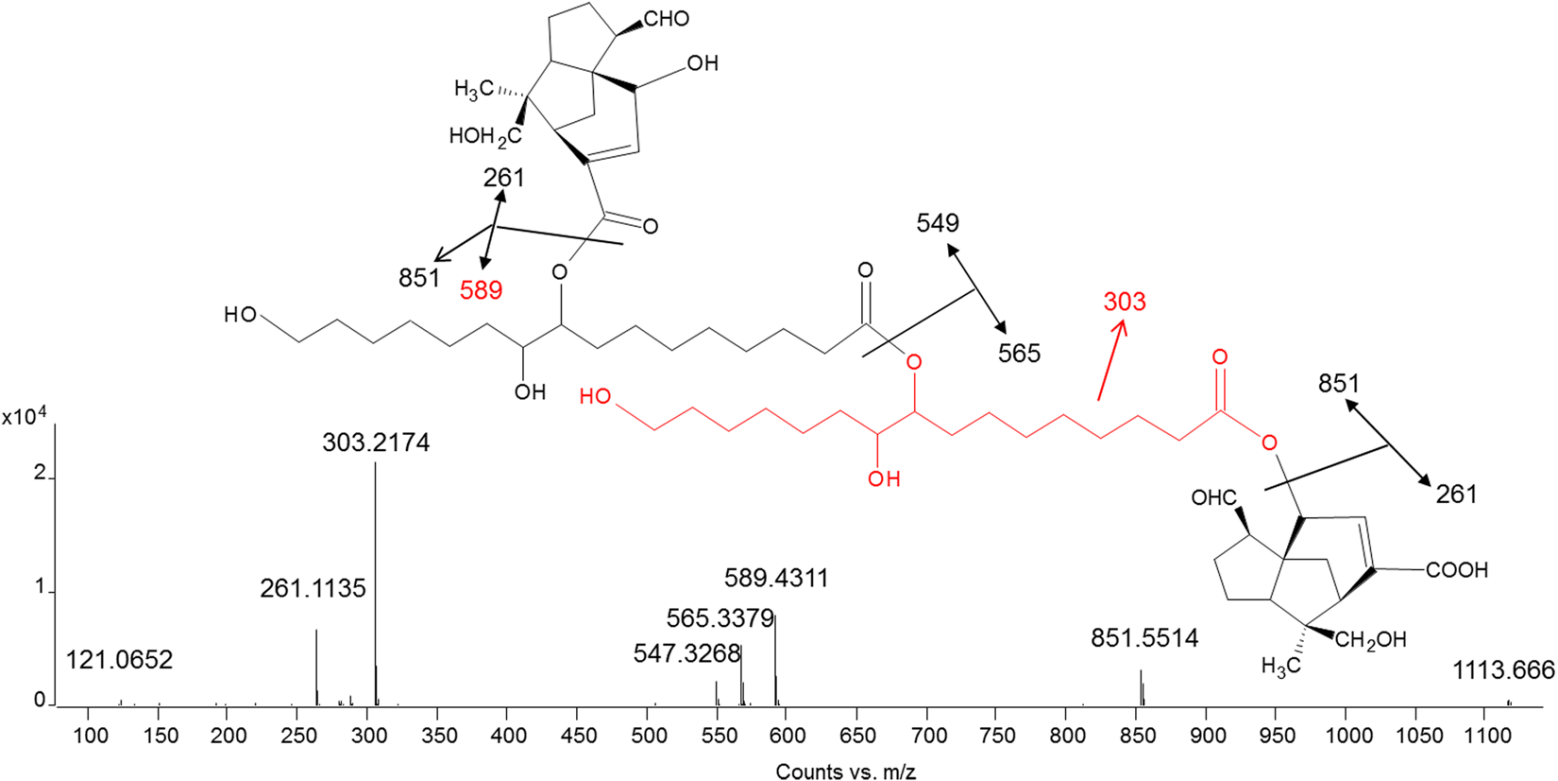
MS/MS spectrum obtained in ESI (−) mode of jalaric-aleuritic-aleuritic-jalaric triester (A-B-B-A configuration).

*Triesters*. MS/MS spectra of triesters showed similar features to those observed for diesters. Three main fragmentations were observed, corresponding to the three ester bonds between the constituting molecules. However, the number of possible configurations dramatically increases. Generally, the higher the level of symmetry of the molecule the simpler the MS/MS spectrum appears, as different fragmentations result in the same fragment ions. However, the same fragment ions can be produced by both subsequent and parallel fragmentation pathways, and this has to be considered when trying to interpret the spectra. The relatively most abundant triester presented two jalaric acid and two aleuritic acid units (Fig. 7). It has been hypothesised that in this ester sesquiterpenoid and aliphatic acids present a A-B-A-B configuration^15,22^, but our MS/MS data indicate that the A-B-B-A configuration is more likely. In fact, the fragment ion with *m/z* 589.4311 (calculated mass 589.4321) is only produced by two esterified aleuritic acids and this was recurrent in the MS/MS spectra of many triesters (Fig. 7, Table 1 and Appendix). Triesters with other configurations, such as A-B-B-C and A-B-C-D were also observed, and for most of them the aliphatic acids occupied the central part of the molecule.

**Figure 8:**
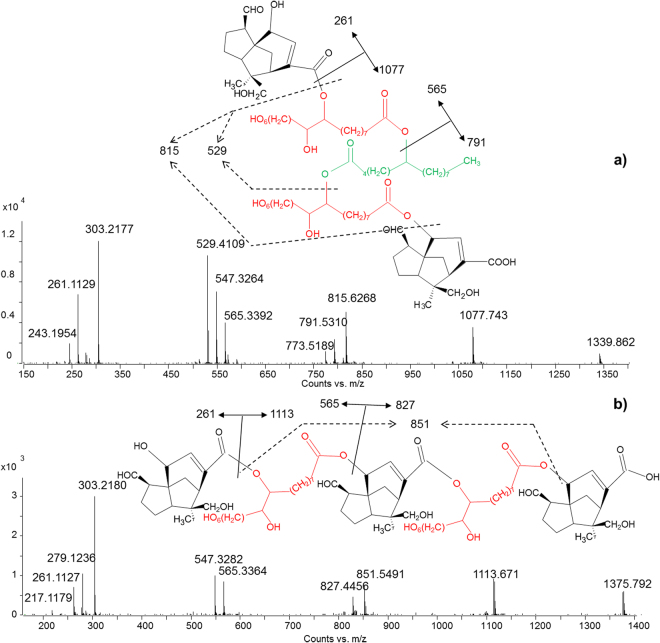
MS/MS spectra obtained in ESI (−) mode of (**a**) jalaric-aleuritic-butolic-aleuritic-jalaric tetraester (A-B-C-B-A configuration) and (**b**) jalaric-aleuritic-jalaric-aleuritic-jalaric tetraester (A-B-A-B-A configuration).

*Tetraesters*. With regards to the tetraesters, two main configurations of molecules were detected: A-B-C-B-A, where A is a sesquiterpenoid acid, B is aleuritic acid and C is butolic acid, and A-B-A-B-A, where A is a sesquiterpenoid acid and B is aleuritic acid. Figure 8 presents the MS/MS spectra of two of these compounds. Based on the observed fragmentation, the first one was ascribed to jalaric-aleuritic-butolic-aleuritic-jalaric tetraester (Fig. 8a), and the second to jalaric-aleuritic-jalaric-aleuritic-jalaric tetraester (Fig. 8b). Also in these cases, some specific fragment ions enabled the order of the constituent acids to be determined. In particular, the fragment ions with *m/z* 565.3392 (calculated mass 565.3382) and 529.4109 (calculate mass 529.4110) derive from jalaric-aleuritic and aleuritic-butolic fragments, respectively, indicating that these must be linked together. The co-presence of fragment ions with *m/z* 791.5310 (calculated mass 791.5315) and 815.6268 (calculated mass 815.6254) generally reveals that jalaric-aleuritic-butolic and aleuritic-butolic-aleuritic fragments are both present in the molecule. Aleuritic-jalaric-aleuritic and jalaric-aleuritic-jalaric fragments produce *m/z* peaks at 851.5526 (calculated mass) and 827.4587 (calculated mass), respectively.

The A-B-B-B-A configuration was also observed to a minor extent, as the jalaric-aleuritic-aleuritic-aleuritic-jalaric tetraester was identified (Table 1, Appendix).

*Other components*. Pentaesters, hexaesters and heptaesters were also detected, but the exact configurations were not ascertained based on MS/MS data interpretation (Appendix). This was sometimes due to the low relative abundances of some *m/z* peaks in the MS/MS spectra. Moreover, as the size of the molecules increases, fragment ions deriving from reconfigurations in the collision cell are also possible and this further complicates the interpretation. For these reasons, the order of the acids used to describe these polyesters in the figures and tables is only hypothetical and needs further confirmation.

It was also interesting to note that butolic acid was frequently found as a polyester component. This suggested that, in addition to its plasticiser function^8^, some structural function might be played by the molecule as well. Interestingly, butolic acid was only found in the odd-number polyesters.

### Historic samples – samples S1 and S2 from the Salvemini collection (Florence)

**Figure 9:**
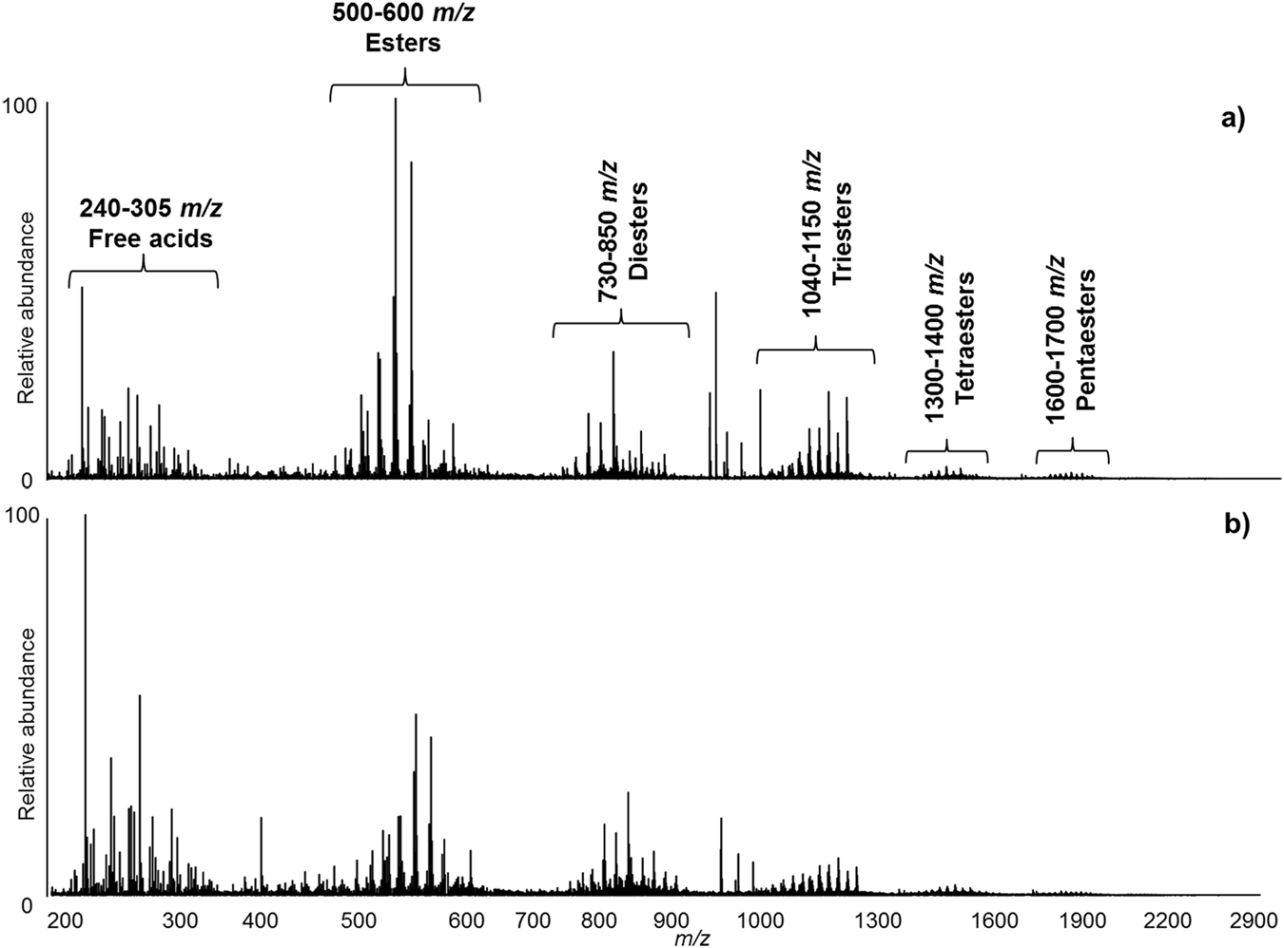
Overall mass spectra obtained by FIA-ESI-Q-ToF of the methanol extracts of samples (**a**) S1 and (**b**) S2 (*m/z* range 200–3000).

The overall mass spectra obtained by FIA analyses of samples S1 and S2 are shown in Fig. 9. In comparing the results to those obtained for sample S0, the main difference observed was the absence of the *m/z* cluster peaks corresponding to polyesters with seven and eight units. Additionally, especially for sample S2, the relative abundances of the *m/z* peaks corresponding to free acids were higher compared to sample S0. This might be interpreted as indicative of the occurrence of hydrolysis in these aged samples, resulting in the partial cleavage of ester bonds and consequent release of free acids. It has to be underlined that, if further esterification and cross-linking had occurred over time, as sometimes suggested in the literature as a possible result of ageing^18,19^, ESI ionisation might not be able to distinguish it. In-source fragmentation of very high molecular weight polymers, in fact, is possible, leading to the formation of fragment ions with the same *m/z* of free acids and oligoesters.

The observations were confirmed by the results obtained by HPLC-ESI-Q-ToF (Figure S1, Supplementary Information). Free acids were relatively more abundant in samples S1 and S2 compared to sample S0, as observed in the first part of the chromatograms. In addition, the peak of butolic acid showed a remarkably high relative abundance in sample S2, as also observed in the FIA results, reflecting a difference in the composition of this sample compared to the others.

A slight increase in the relative abundance of shellolic acid, laccishelloic acid and related compounds was observed in samples S1 and S2 in comparison with sample S0. This has already been reported as a common finding in aged shellac, resulting from the oxidation of jalaric and laccijalaric acids^6^. The oxidised compounds with a keto group identified in sample S0 (Fig. 4) showed higher relative abundances in sample S2 compared to samples S0 and S1, whereas they showed lower relative abundances in sample S1 compared to sample S0. Therefore, the presence of these oxidised products does not seem to be related only to the natural ageing of the resin, but is also likely to reflect differences in the natural composition of the resin, or different treatments undergone by the original sticklac to produce the shellac.

## Conclusions

The combined use of FIA-ESI-Q-ToF and HPLC-ESI-Q-ToF proved to be a powerful strategy for the molecular characterisation of shellac resin. The abilities of Q-ToF to provide high resolution mass measurements and the acquisition of highly informative tandem mass spectra enabled the main acid components of shellac (sesquiterpenoid and polyhydroxyaliphatic acids) to be identified, and minor sesquiterpenoid and polyhydroxyaliphatic acids to be detected and their molecular structures to be elucidated or hypothesised. In addition, esters and polyesters constituted by up to eight acid units were also detected and their structures, when possible, suggested, discussing the way sesquiterpenoids and polyhydroxyaliphatic acids are linked to one another. The possibility to investigate the constituting polyesters and their structures represents a major advancement with respect to the most commonly applied techniques (GC-MS and Py-GC-MS), which require the cleavage of the ester bonds, thus completely losing any structural information.

Some compounds were also detected for the first time, and the MS data interpretation revealed that these resulted from the oxidation of hydroxyl moieties of aleuritic, butolic, jalaric, laccijalaric and shellolic acids. Naturally aged historic shellac samples showed different profiles in terms of relative abundances of free acids and their oxidation products. FIA-ESI-Q-ToF data suggested that macromolecular components are significantly affected by ageing, mainly in terms of hydrolysis of ester bonds.

This work represents a first methodological advance in the application of HPLC-MS to the characterisation of shellac resin and further developments can be achieved, especially considering the wide-ranging applications of this material. In addition, the method can be potentially applied to the characterisation of other natural resins, possibly providing significant additional information.

## Materials and Methods

### Chemicals and reagents

Methanol (Sigma Aldrich, HPLC grade, purity ≥ 99.9%), acetonitrile (VWR, HiPerSolv CHROMANORM, HPLC grade, purity ≥ 99.9%), formic acid (Sigma Aldrich, eluent additive for LC-MS) and aleuritic acid (Alfa Aesar, purity 95%) were used as received.

### Samples

Three samples of shellac were analysed. A relatively fresh shellac sample (S0) from the British Museum (BM) reference collection (BMR No. REFC-107-T) was used as reference material. The material was stored in a flask in the laboratory environment.

Two samples of historic shellac, dating back to the early 19^th^ century (samples S1 and S2), were provided by the natural history collection of the Salvemini Collection in Florence. Two small flakes of pure material were examined. Sample S1 was taken from a piece of shellac from East India. The provenance of sample S2 is not known, although it is supposed to come from East Asian Portuguese colonies.

A reference sample of aleuritic acid was also analysed by HPLC-ESI-Q-ToF.

### Sample preparation

Samples (*ca*. 0.5 mg) were dissolved in 400 µL of methanol by heating them at 40 °C for 1 h. Complete dissolution of the sample was observed, in accordance with the literature^18^. After centrifugation, the supernatant was transferred to a fresh 250 µL insert and 10–20 µL of the solution were analysed.

#### FIA-ESI-Q-ToF

Aliquots (20 µL) of the methanol extracts were injected into the FIA-ESI-Q-ToF system with an eluent mixture of 90% acetonitrile with 0.1% formic acid and 10% water with 0.1% formic acid at 0.4 mL/min flow rate.

Analyses were carried out using a 1260 Infinity HPLC (Agilent Technologies), coupled to a 1100 DAD detector (Hewlett-Packard) and a Quadrupole-Time of Flight tandem mass spectrometer 6530 Infinity Q-ToF detector (Agilent Technologies) by a Jet Stream ESI interface (Agilent Technologies).

The ESI was operated in negative mode and the experimental conditions were: drying gas (N_2_, purity > 98%): 350 °C and 10 L/min; capillary voltage 4.0 KV; nebulizer gas 40 psig; sheath gas (N_2_, purity > 98%): 375 °C and 11 L/min.

High resolution MS data were acquired in the range 100–3000 *m/z*. The fragmentor was kept at 150 V, nozzle voltage 1000 V, skimmer 65 V, octapole RF 750 V.

#### HPLC-ESI-Q-ToF

Aliquots of the methanol extracts were analysed by HPLC-ESI-Q-ToF. Experimental conditions were: Zorbax Extend-C18 column (2.1 mm × 50 mm, 1.8 μm particle size); 0.4 mL/min flow rate; 10 μL injection volume for MS experiments and 20 μL for MS/MS experiments; 40 °C column temperature. Separation was achieved using a gradient of water with 0.1% formic acid (eluent A) and acetonitrile with 0.1% formic acid (eluent B). The elution gradient was programmed as follows: initial conditions 95% A, followed by a linear gradient to 100% B in 10 min, and then held for 2 min. Re-equilibration time for each analysis was 10 min.

ESI conditions and acquisition parameters of MS data were the same described for FIA analyses. For the MS/MS experiments, different voltages in the collision cell were tested for Collision Induced Dissociation (CID), in order to maximise the information obtained from the fragmentation. The collision gas was nitrogen (purity 99.999%). The data were collected by targeted MS/MS acquisition with an MS scan rate of 1.0 spectra/sec and an MS/MS scan rate of 1.0 spectra/sec. MassHunter® Workstation Software was used to carry out mass spectrometer control, data acquisition, and data analysis.

### Data availability

All data generated or analysed during this study are included in this published article (and its Supplementary Information files).

## Electronic supplementary material

Supplementary information
