# Supplementary material for: The characterisation of shellac resin by flow injection and liquid chromatography coupled with electrospray ionisation and mass spectrometry
Source: Sci Rep. 2017 Nov 1;7:14784. doi: 10.1038/s41598-017-14907-7 (PMC5665916; doi:10.1038/s41598-017-14907-7)

The characterisation of shellac resin by flow injection and liquid chromatography coupled with electrospray ionisation and mass spectrometry

Diego Tamburini[1]*, Joanne Dyer[1], Ilaria Bonaduce[2]

[1] Department of Scientific Research, The British Museum, Great Russell Street, WC1B 3DG, London, United Kingdom

[2] Department of Chemistry and Industrial Chemistry, University of Pisa, via G. Moruzzi 13, I-56124, Pisa, Italy

Figure S1. Total Ion Chromatograms (TICs) obtained by HPLC-ESI-Q-ToF analysis of the methanol extracts of samples **a)** S0, **b)** S1 and **c)** S2. Labels refer to Table 1.

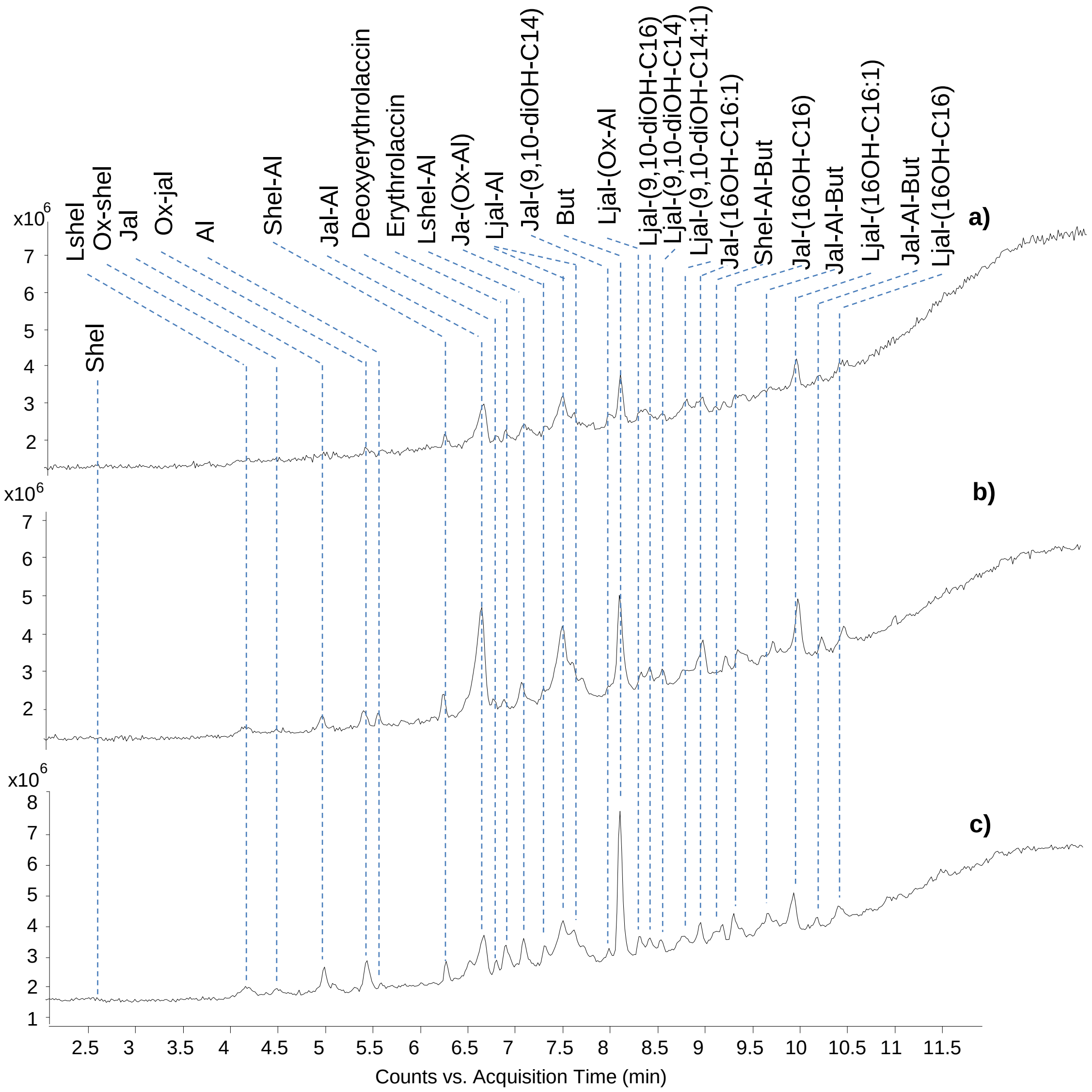

Figure S2. Comparison between chromatographic (top) and MS data (bottom) obtained for the reference of aleuritic acid (a) and the molecule identified in sample S0 (b), showing confirmation of the identification.

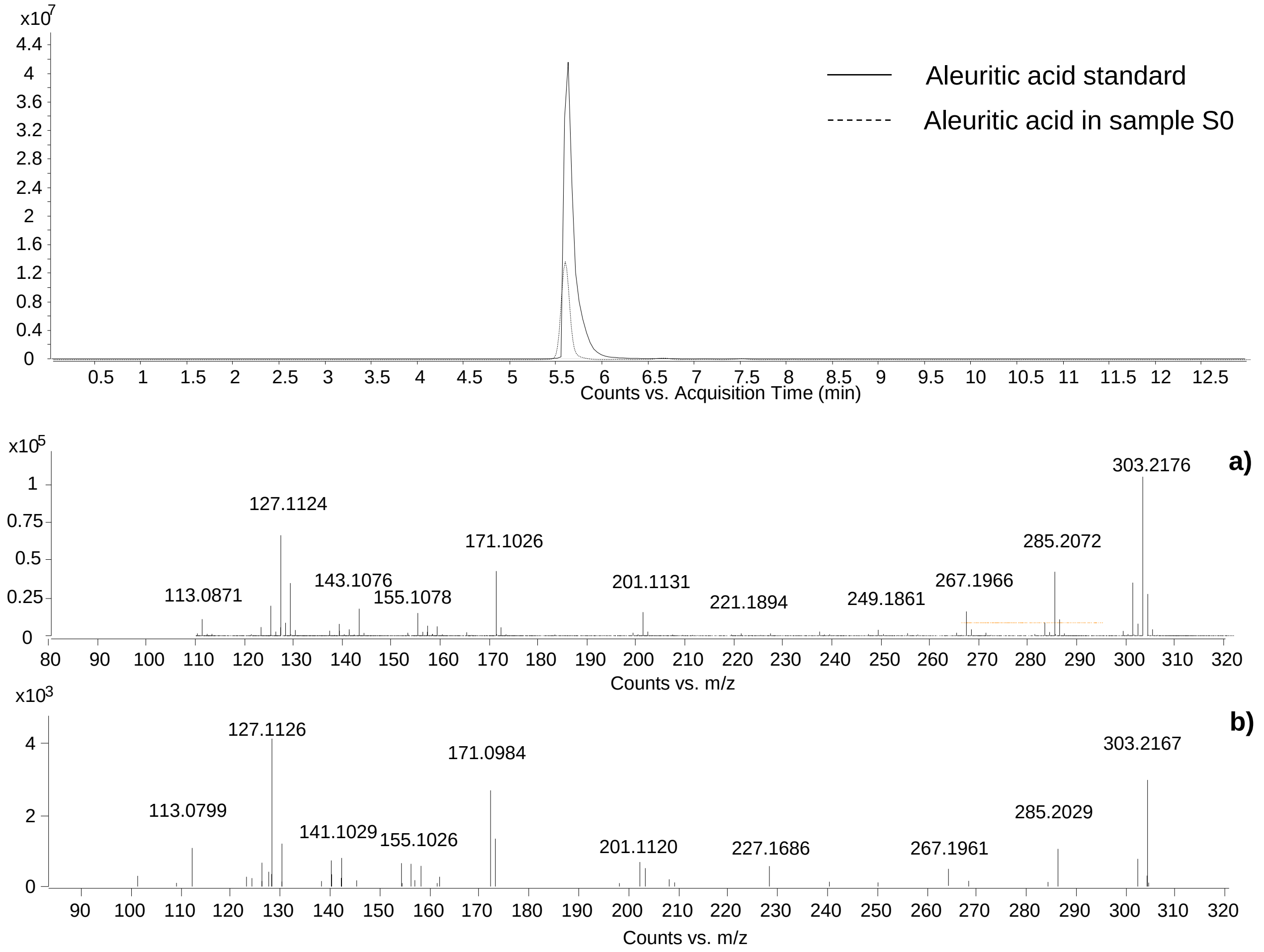

Appendix - MS/MS spectra of shellac components acquired in negative ionisation mode

Variable collision energies in the range 20-40 V were adopted, mainly depending on the molecular weight of the molecules.

6-oxotetradecanoic acid

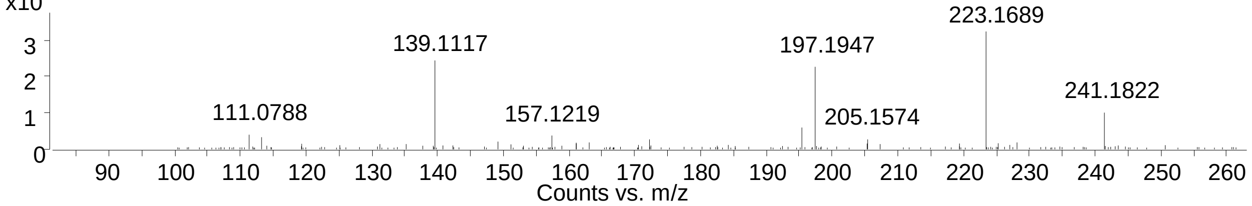

butolic acid (6-hydroxytetradecanoic acid)

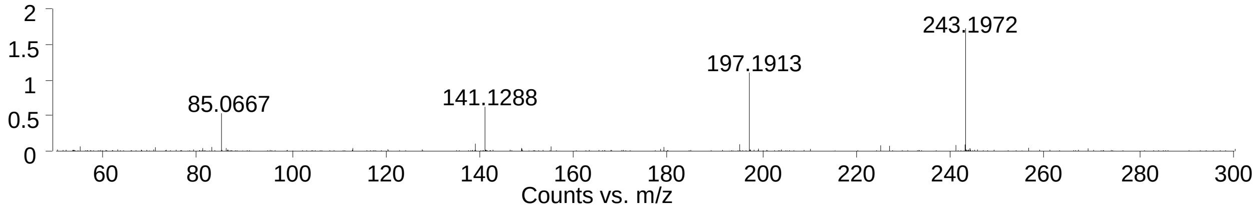

9,10-dihydroxytetradecanoic acid

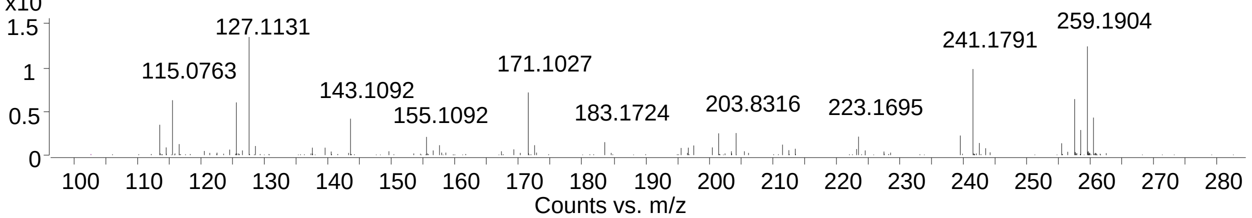

Oxidised laccijalaric acid

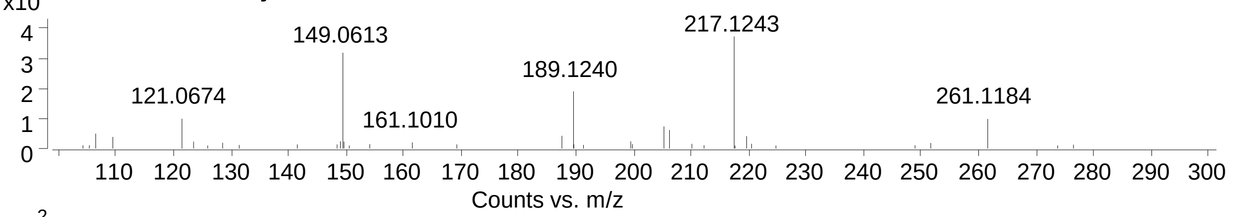

Laccijalaric acid

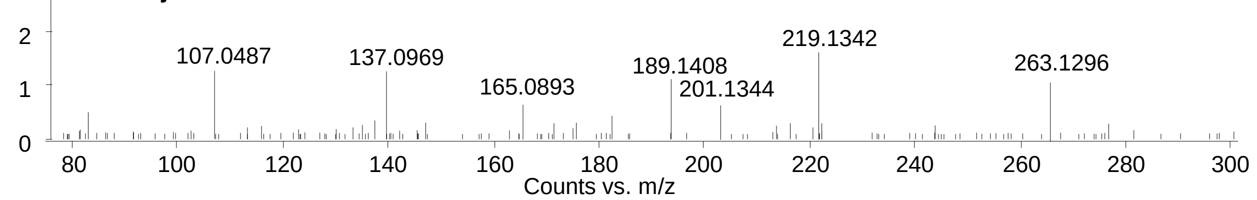

Laccilaksholic acid

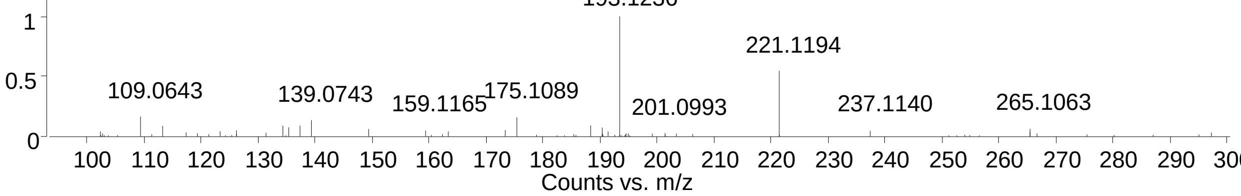

16-hydroxyhexadec-9-enoic acid

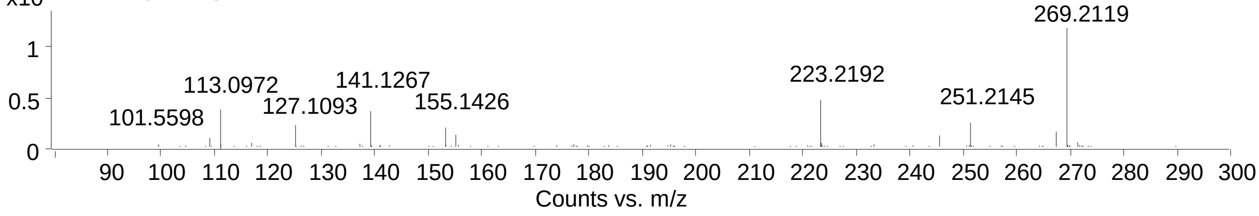

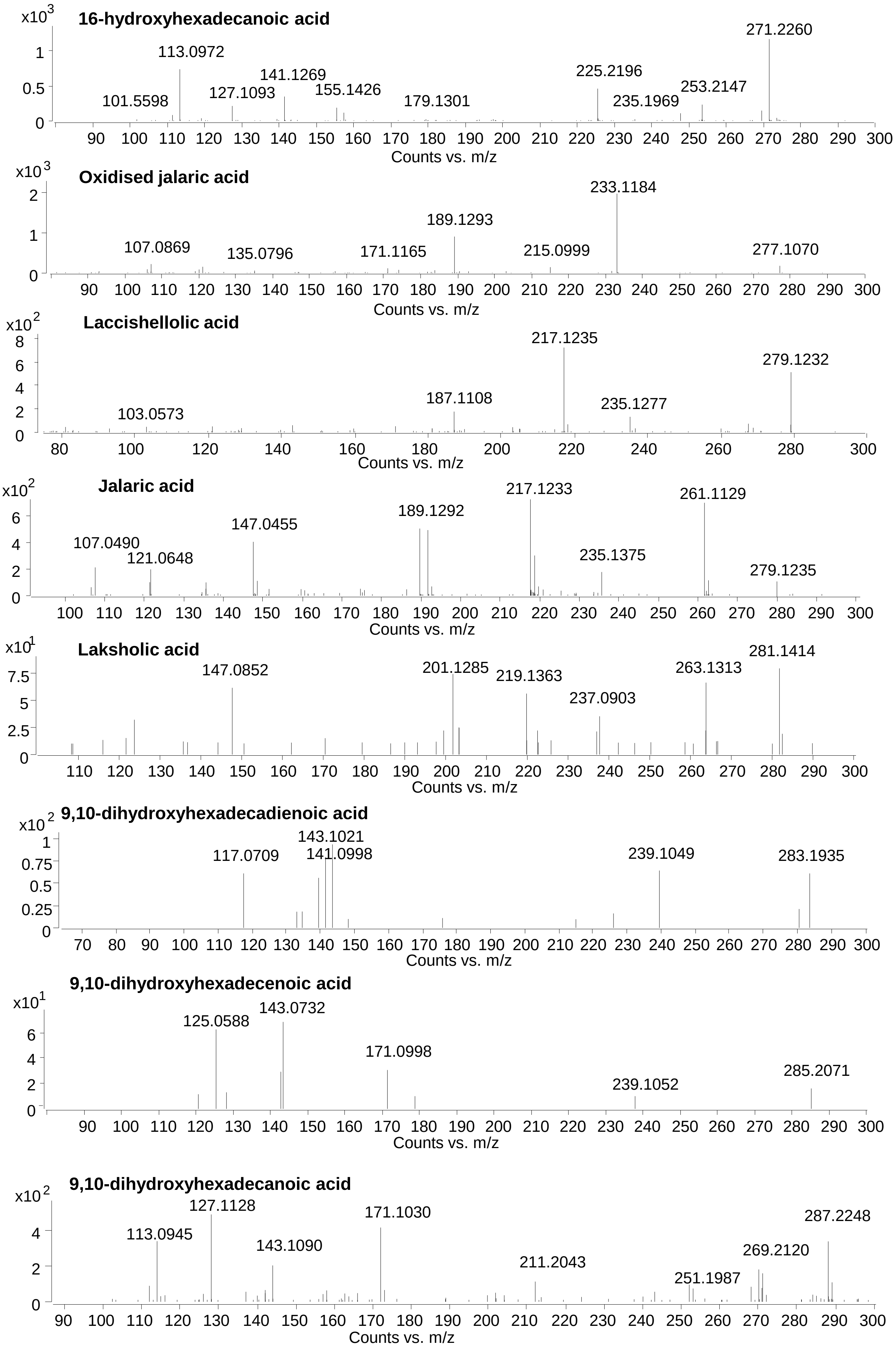
16-hydroxyhexadecanoic acid
x10^3
113.0972
101.5598
127.1093
141.1269
155.1426
179.1301
225.2196
235.1969
253.2147
271.2260
Counts vs. m/z
Oxidised jalaric acid
x10^3
107.0869
135.0796
171.1165
189.1293
215.0999
233.1184
277.1070
Counts vs. m/z
Laccishellolic acid
x10^2
103.0573
187.1108
217.1235
235.1277
279.1232
Counts vs. m/z
Jalaric acid
x10^2
107.0490
121.0648
147.0455
189.1292
217.1233
235.1375
261.1129
279.1235
Counts vs. m/z
Laksholic acid
x10^1
147.0852
201.1285
219.1363
237.0903
263.1313
281.1414
Counts vs. m/z
9,10-dihydroxyhexadecadienoic acid
x10^2
117.0709
143.1021
141.0998
239.1049
283.1935
Counts vs. m/z
9,10-dihydroxyhexadecenoic acid
x10^1
125.0588
143.0732
171.0998
239.1052
285.2071
Counts vs. m/z
9,10-dihydroxyhexadecanoic acid
x10^2
113.0945
127.1128
143.1090
171.1030
211.2043
251.1987
269.2120
287.2248
Counts vs. m/z

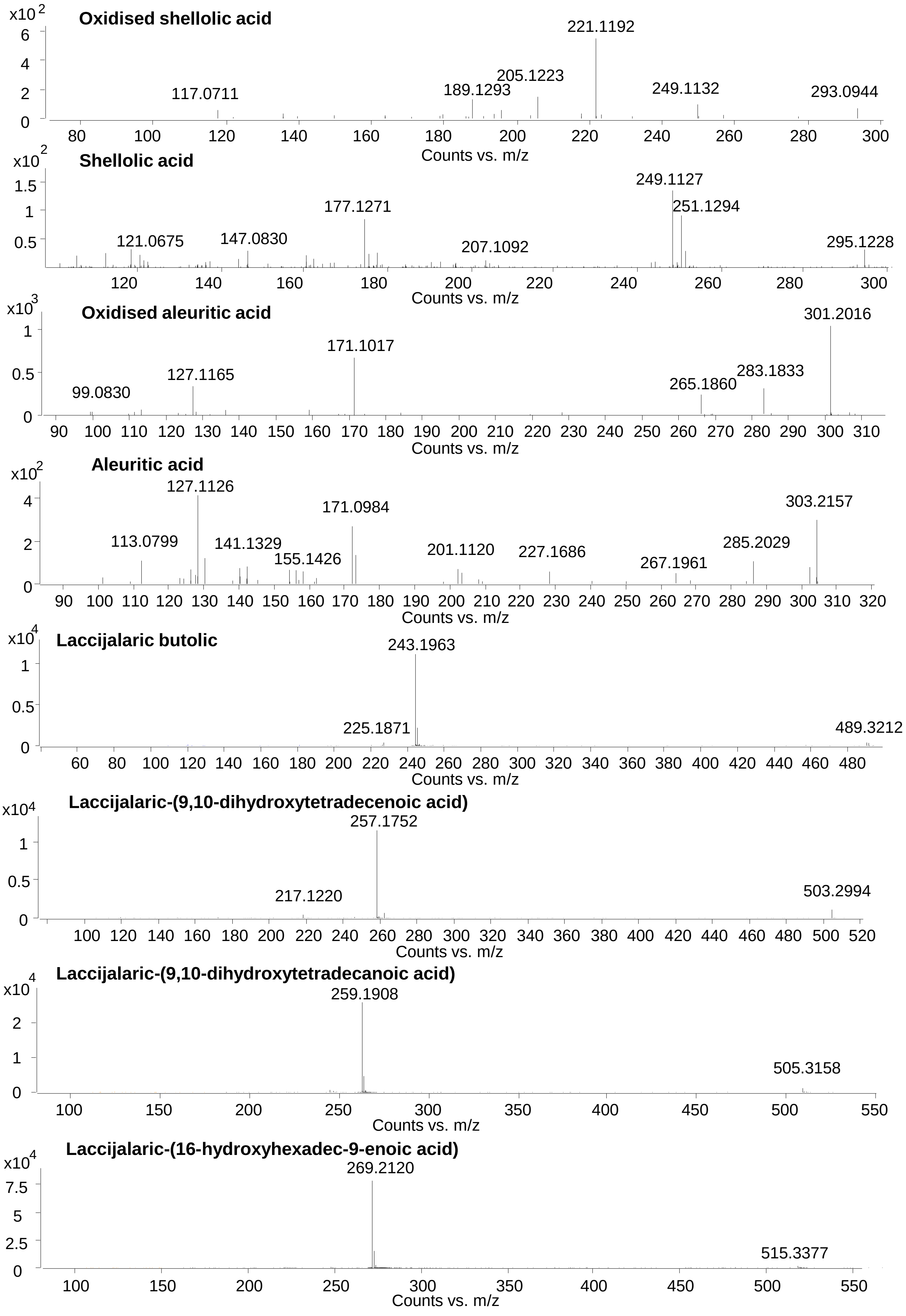
Oxidised shellolic acid
x10 2
221.1192
205.1223
189.1293
117.0711
249.1132
293.0944
Counts vs. m/z
Shellolic acid
x10 2
249.1127
251.1294
177.1271
147.0830
121.0675
207.1092
295.1228
Counts vs. m/z
Oxidised aleuritic acid
x10 3
301.2016
171.1017
127.1165
99.0830
265.1860
283.1833
Counts vs. m/z
Aleuritic acid
x10 2
127.1126
171.0984
303.2157
113.0799
141.1329
155.1426
201.1120
227.1686
285.2029
267.1961
Counts vs. m/z
Laccijalaric butolic
x10 4
243.1963
225.1871
489.3212
Counts vs. m/z
Laccijalaric-(9,10-dihydroxytetradecenoic acid)
x10 4
257.1752
217.1220
503.2994
Counts vs. m/z
Laccijalaric-(9,10-dihydroxytetradecanoic acid)
x10 4
259.1908
505.3158
Counts vs. m/z
Laccijalaric-(16-hydroxyhexadec-9-enoic acid)
x10 4
269.2120
515.3377
Counts vs. m/z

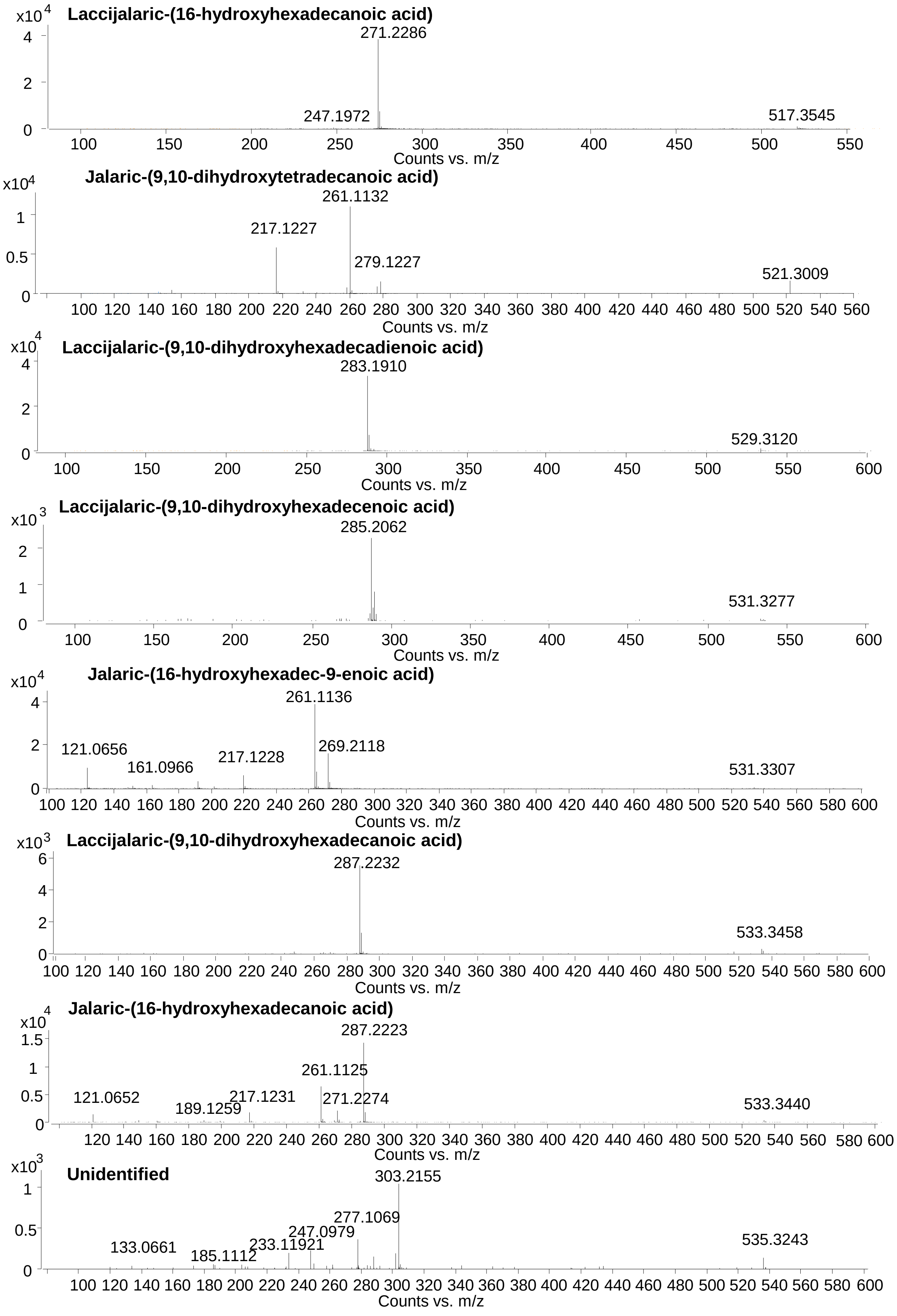

Laccijalaric-(16-hydroxyhexadecanoic acid)
x10^4
271.2286
247.1972
517.3545
Counts vs. m/z
Jalaric-(9,10-dihydroxytetradecanoic acid)
x10^4
261.1132
217.1227
279.1227
521.3009
Counts vs. m/z
Laccijalaric-(9,10-dihydroxyhexadecadienoic acid)
x10^4
283.1910
529.3120
Counts vs. m/z
Laccijalaric-(9,10-dihydroxyhexadecenoic acid)
x10^3
285.2062
531.3277
Counts vs. m/z
Jalaric-(16-hydroxyhexadec-9-enoic acid)
x10^4
261.1136
121.0656
269.2118
161.0966
217.1228
531.3307
Counts vs. m/z
Laccijalaric-(9,10-dihydroxyhexadecanoic acid)
x10^3
287.2232
533.3458
Counts vs. m/z
Jalaric-(16-hydroxyhexadecanoic acid)
x10^4
287.2223
261.1125
121.0652
217.1231
271.2274
189.1259
533.3440
Counts vs. m/z
Unidentified
x10^3
303.2155
277.1069
247.0979
233.11921
133.0661
185.1112
535.3243
Counts vs. m/z

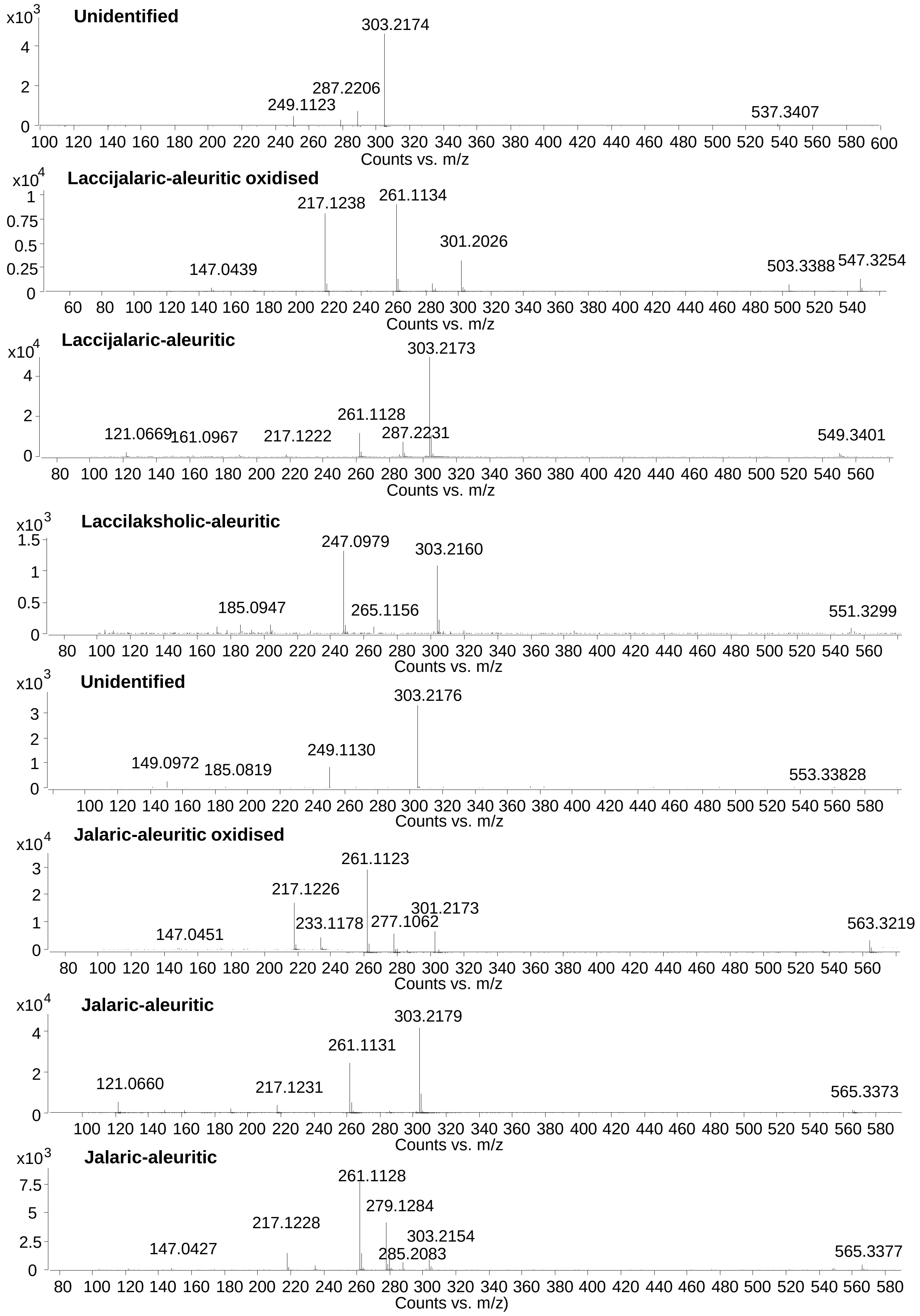
Unidentified
x10^3
303.2174
287.2206
249.1123
537.3407
Counts vs. m/z
Laccijalaric-aleuritic oxidised
x10^4
217.1238
261.1134
301.2026
147.0439
503.3388
547.3254
Counts vs. m/z
Laccijalaric-aleuritic
x10^4
303.2173
261.1128
121.0669
161.0967
217.1222
287.2231
549.3401
Counts vs. m/z
Laccilaksholic-aleuritic
x10^3
247.0979
303.2160
185.0947
265.1156
551.3299
Counts vs. m/z
Unidentified
x10^3
303.2176
249.1130
149.0972
185.0819
553.33828
Counts vs. m/z
Jalaric-aleuritic oxidised
x10^4
261.1123
217.1226
301.2173
233.1178
277.1062
147.0451
563.3219
Counts vs. m/z
Jalaric-aleuritic
x10^4
303.2179
261.1131
121.0660
217.1231
565.3373
Counts vs. m/z
Jalaric-aleuritic
x10^3
261.1128
279.1284
217.1228
303.2154
147.0427
285.2083
565.3377
Counts vs. m/z)

Laccishellolic-aleuritic

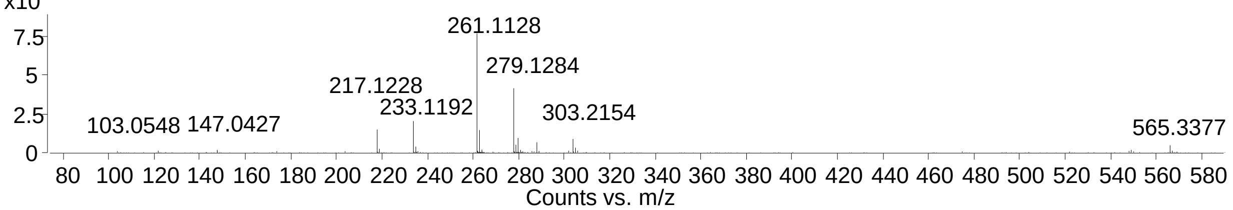

Laksholic-aleuritic

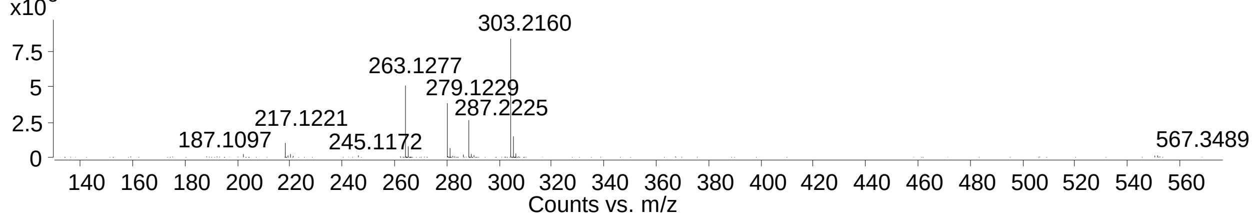

Shellolic-aleuritic oxidised

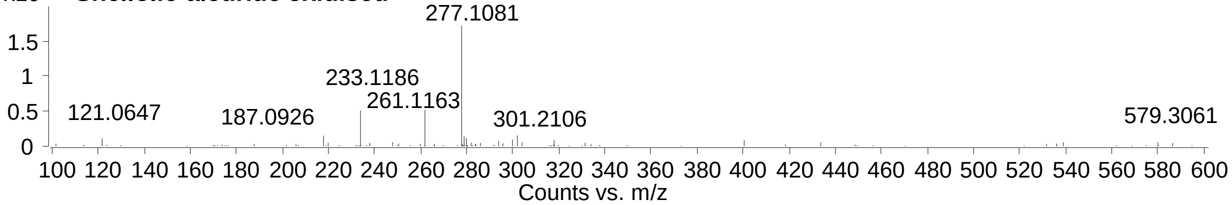

Shellolic-aleuritic

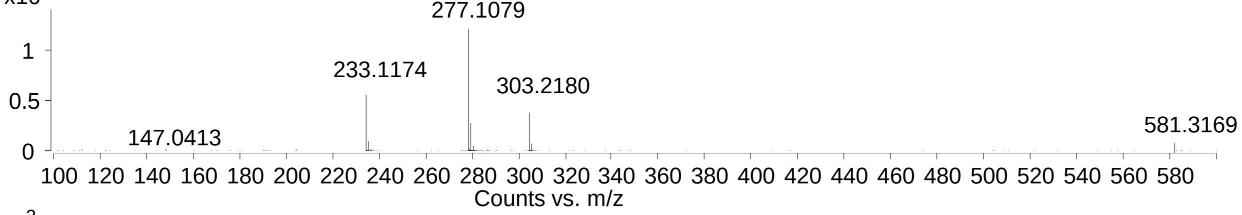

Laccijalaric-(9,10-dihydroxytetradecanoic)-butolic

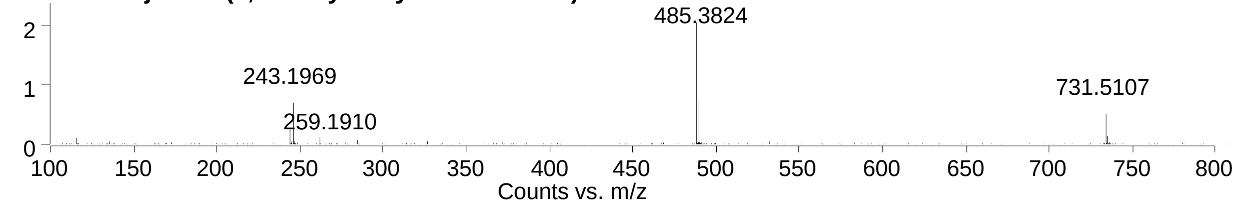

Laccijalaric-(16-hydroxyhexadec-9-enoic)-butolic

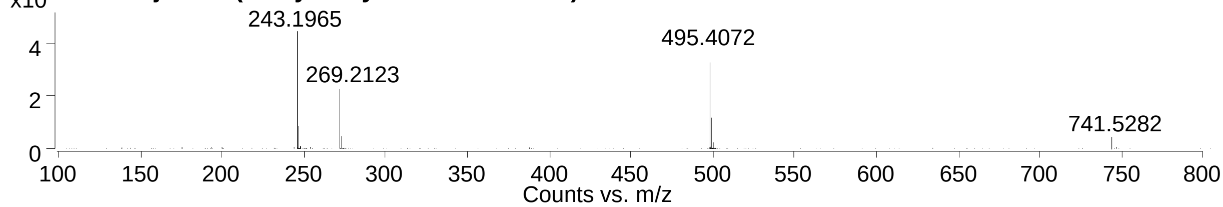

Laccijalaric-(9,10-dihydroxyhexadecadienoic)-butolic

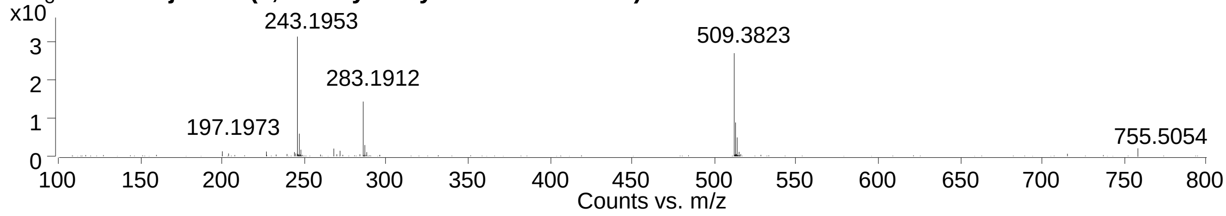

Jalaric-(16-hydroxyhexadec-9-enoic)-butolic

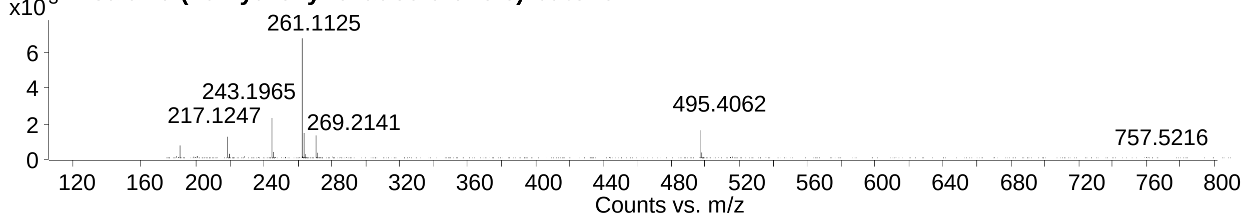

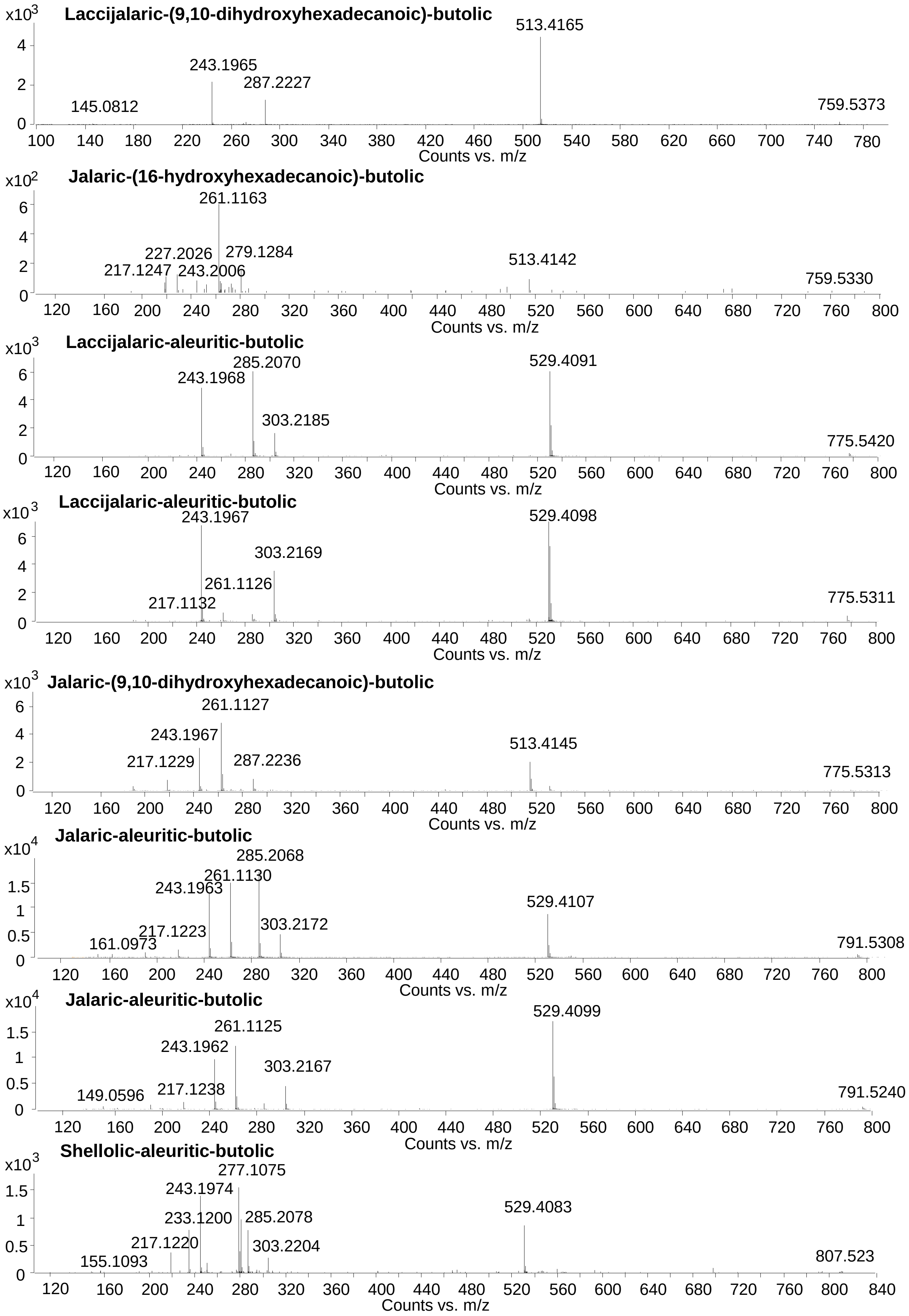
Laccijalaric-(9,10-dihydroxyhexadecanoic)-butolic
x10^3
513.4165
243.1965
287.2227
145.0812
759.5373
Counts vs. m/z
Jalaric-(16-hydroxyhexadecanoic)-butolic
x10^2
261.1163
227.2026
279.1284
217.1247
243.2006
513.4142
759.5330
Counts vs. m/z
Laccijalaric-aleuritic-butolic
x10^3
285.2070
243.1968
529.4091
303.2185
775.5420
Counts vs. m/z
Laccijalaric-aleuritic-butolic
x10^3
243.1967
529.4098
303.2169
261.1126
217.1132
775.5311
Counts vs. m/z
Jalaric-(9,10-dihydroxyhexadecanoic)-butolic
x10^3
261.1127
243.1967
513.4145
217.1229
287.2236
775.5313
Counts vs. m/z
Jalaric-aleuritic-butolic
x10^4
285.2068
261.1130
243.1963
529.4107
217.1223
303.2172
161.0973
791.5308
Counts vs. m/z
Jalaric-aleuritic-butolic
x10^4
529.4099
261.1125
243.1962
303.2167
149.0596
217.1238
791.5240
Counts vs. m/z
Shellolic-aleuritic-butolic
x10^3
277.1075
243.1974
233.1200
285.2078
529.4083
217.1220
303.2204
155.1093
807.523
Counts vs. m/z

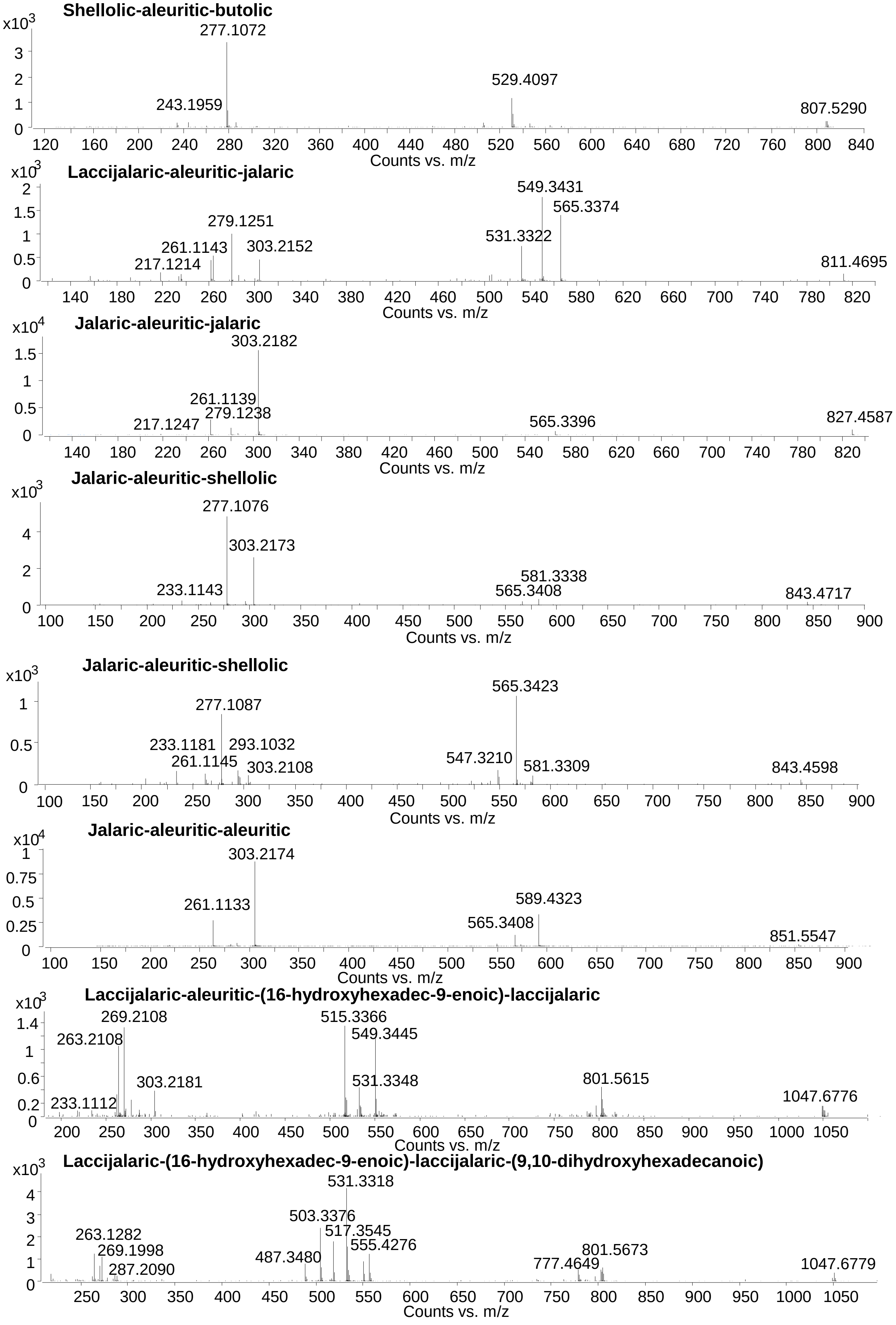
Shellolic-aleuritic-butolic
x10^3
277.1072
243.1959
529.4097
807.5290
Counts vs. m/z
Laccijalaric-aleuritic-jalaric
x10^3
549.3431
565.3374
279.1251
531.3322
261.1143
303.2152
217.1214
811.4695
Counts vs. m/z
Jalaric-aleuritic-jalaric
x10^4
303.2182
261.1139
279.1238
217.1247
565.3396
827.4587
Counts vs. m/z
Jalaric-aleuritic-shellolic
x10^3
277.1076
303.2173
581.3338
233.1143
565.3408
843.4717
Counts vs. m/z
Jalaric-aleuritic-shellolic
x10^3
565.3423
277.1087
233.1181
293.1032
261.1145
303.2108
547.3210
581.3309
843.4598
Counts vs. m/z
Jalaric-aleuritic-aleuritic
x10^4
303.2174
261.1133
589.4323
565.3408
851.5547
Counts vs. m/z
Laccijalaric-aleuritic-(16-hydroxyhexadec-9-enoic)-laccijalaric
x10^3
269.2108
515.3366
263.2108
549.3445
303.2181
531.3348
801.5615
233.1112
1047.6776
Counts vs. m/z
Laccijalaric-(16-hydroxyhexadec-9-enoic)-laccijalaric-(9,10-dihydroxyhexadecanoic)
x10^3
531.3318
503.3376
517.3545
263.1282
555.4276
269.1998
487.3480
801.5673
777.4649
287.2090
1047.6779
Counts vs. m/z

Laccijalaric-aleuritic-jalaric-(16-hydroxyhexadec-9-enoic)

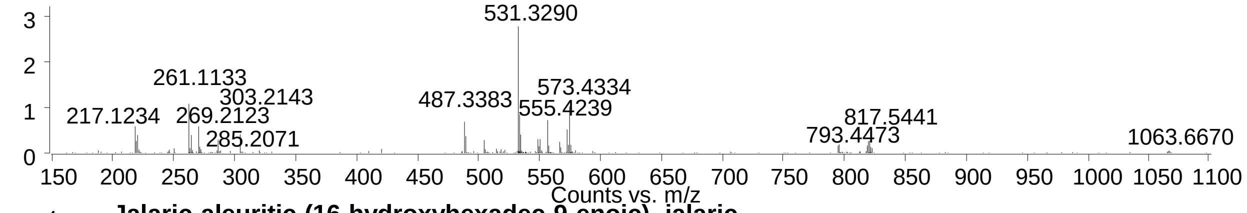

Jalaric-aleuritic-(16-hydroxyhexadec-9-enoic)- jalaric

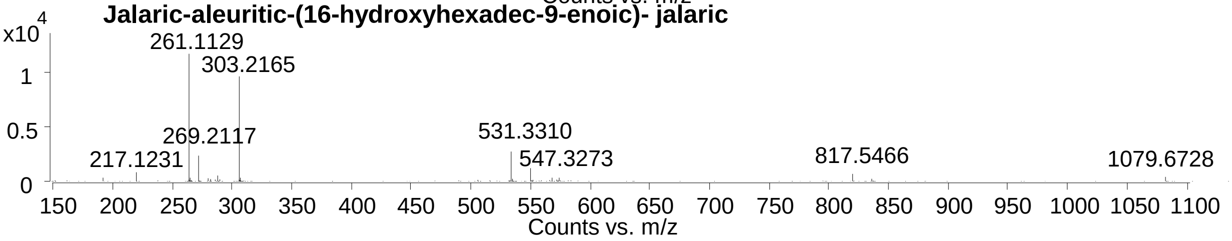

Laccijalaric-aleuritic-(9,10-dihydroxyhexadecenoic)-jalaric

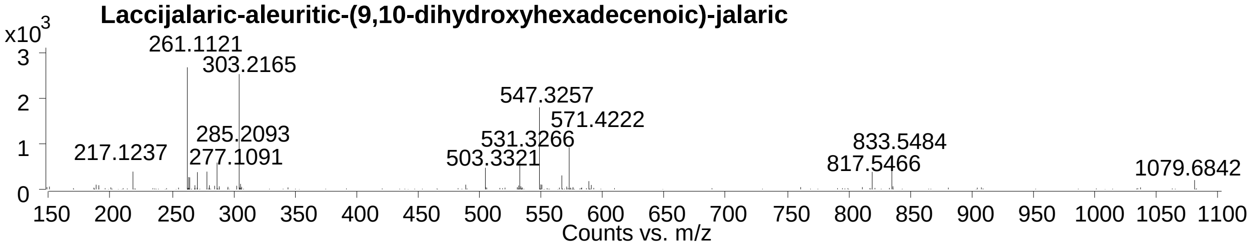

Laccijalaric-aleuritic-aleuritic-laccijalaric

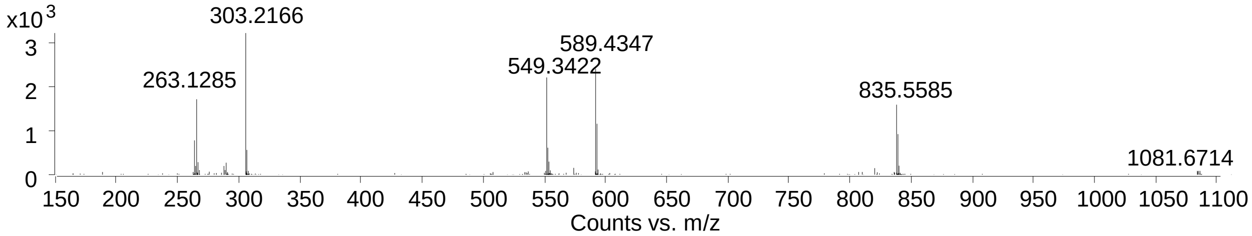

Jalaric-aleuritic-(9,10-dihydroxyhexadecenoic)-jalaric

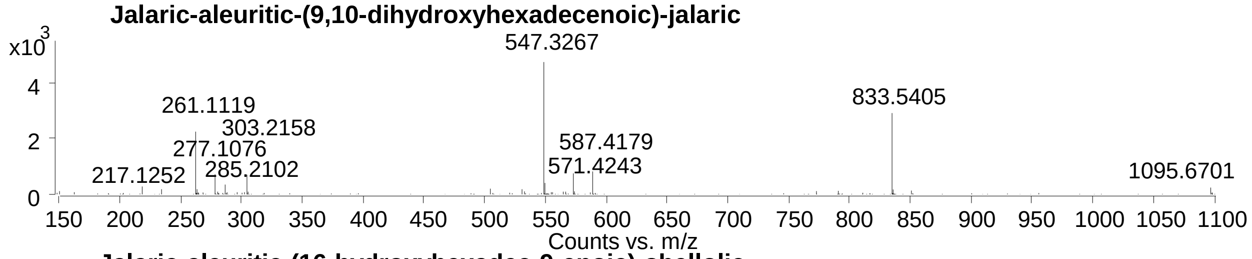

Jalaric-aleuritic-(16-hydroxyhexadec-9-enoic)-shellolic

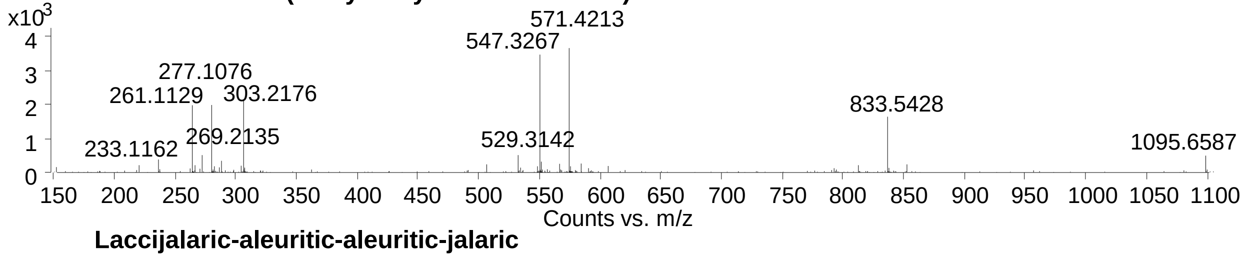

Laccijalaric-aleuritic-aleuritic-jalaric

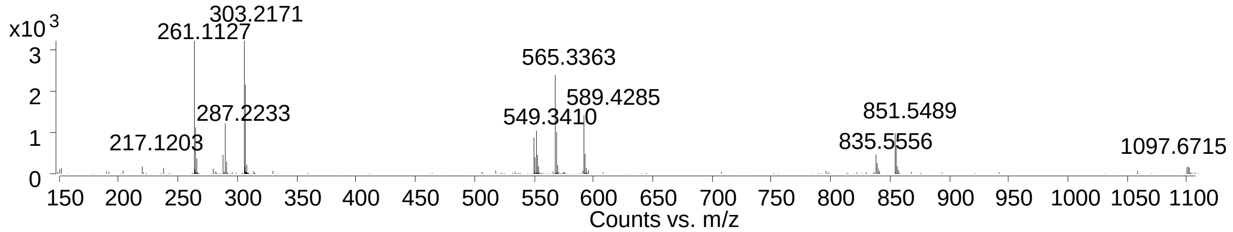

Laccijalaric-aleuritic-aleuritic-jalaric

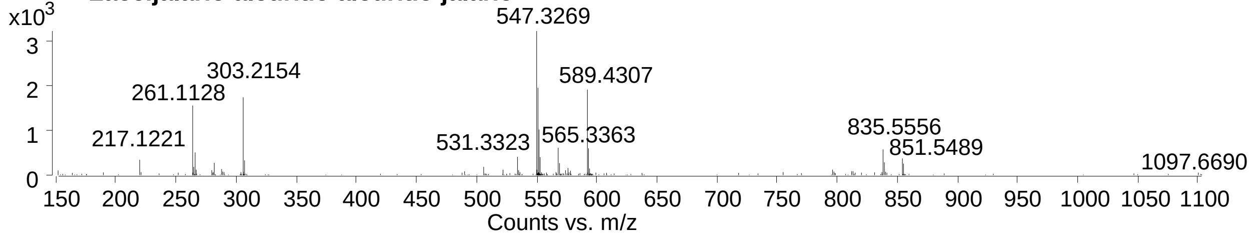

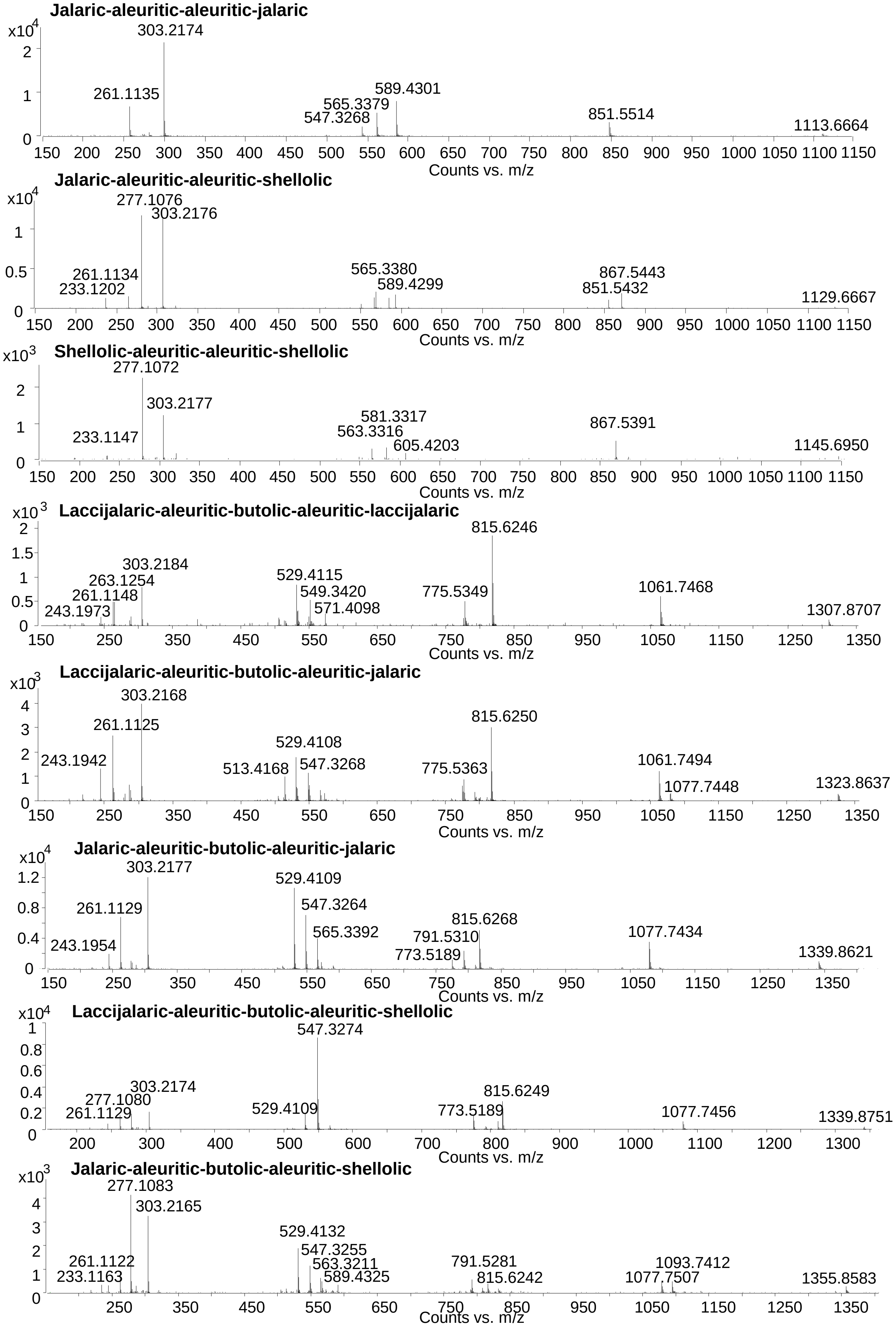

Jalaric-aleuritic-aleuritic-jalaric
x10^4
303.2174
261.1135
589.4301
565.3379
547.3268
851.5514
1113.6664
Counts vs. m/z
Jalaric-aleuritic-aleuritic-shellolic
x10^4
277.1076
303.2176
261.1134
233.1202
565.3380
589.4299
867.5443
851.5432
1129.6667
Counts vs. m/z
Shellolic-aleuritic-aleuritic-shellolic
x10^3
277.1072
303.2177
233.1147
581.3317
563.3316
605.4203
867.5391
1145.6950
Counts vs. m/z
Laccijalaric-aleuritic-butolic-aleuritic-laccijalaric
x10^3
815.6246
303.2184
263.1254
261.1148
243.1973
529.4115
549.3420
571.4098
775.5349
1061.7468
1307.8707
Counts vs. m/z
Laccijalaric-aleuritic-butolic-aleuritic-jalaric
x10^3
303.2168
261.1125
243.1942
815.6250
529.4108
513.4168
547.3268
775.5363
1061.7494
1077.7448
1323.8637
Counts vs. m/z
Jalaric-aleuritic-butolic-aleuritic-jalaric
x10^4
303.2177
261.1129
243.1954
529.4109
547.3264
565.3392
815.6268
791.5310
773.5189
1077.7434
1339.8621
Counts vs. m/z
Laccijalaric-aleuritic-butolic-aleuritic-shellolic
x10^4
547.3274
303.2174
277.1080
261.1129
529.4109
815.6249
773.5189
1077.7456
1339.8751
Counts vs. m/z
Jalaric-aleuritic-butolic-aleuritic-shellolic
x10^3
277.1083
303.2165
261.1122
233.1163
529.4132
547.3255
563.3211
589.4325
791.5281
815.6242
1093.7412
1077.7507
1355.8583
Counts vs. m/z

Laccijalaric-aleuritic-jalaric-aleuritic-jalaric

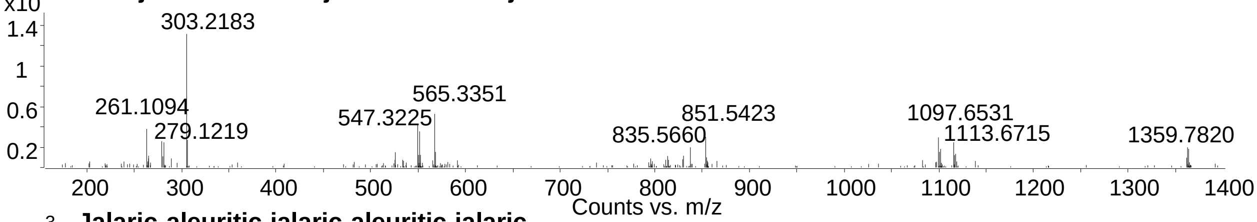

Jalaric-aleuritic-jalaric-aleuritic-jalaric

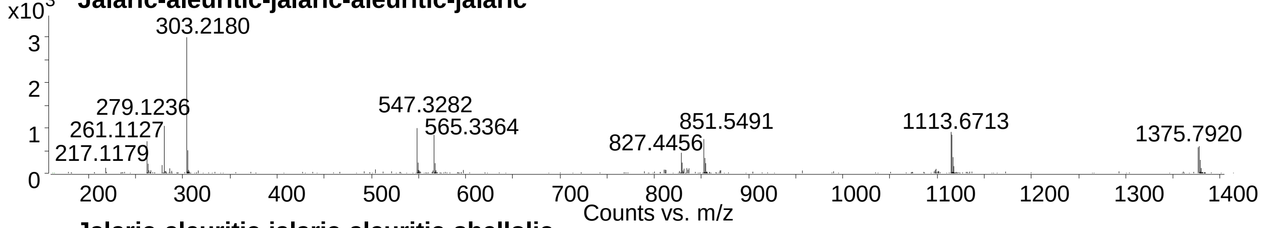

Jalaric-aleuritic-jalaric-aleuritic-shellolic

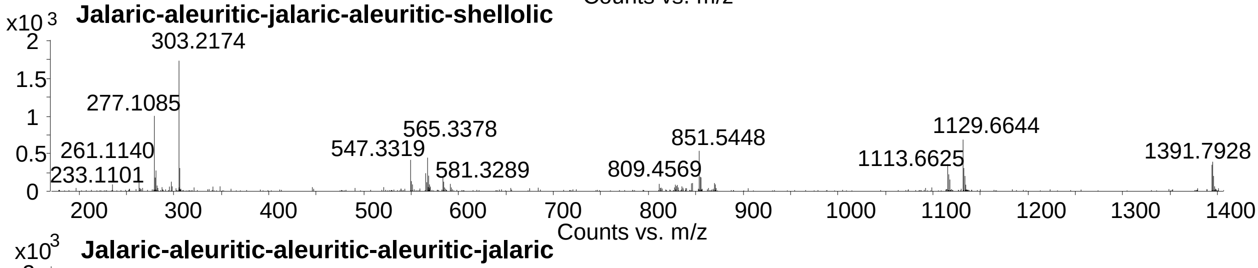

Jalaric-aleuritic-aleuritic-aleuritic-jalaric

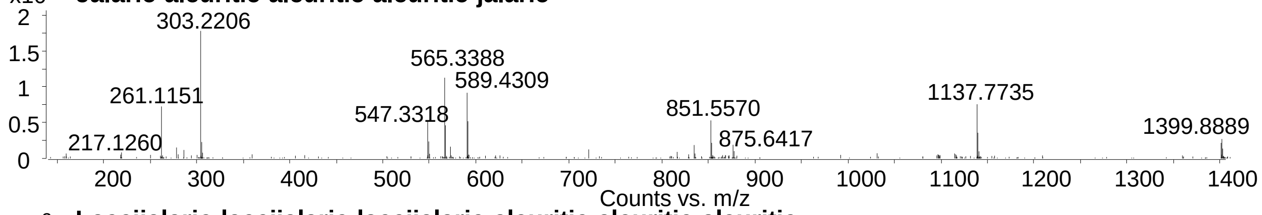

Laccijalaric-laccijalaric-laccijalaric-aleuritic-aleuritic-aleuritic

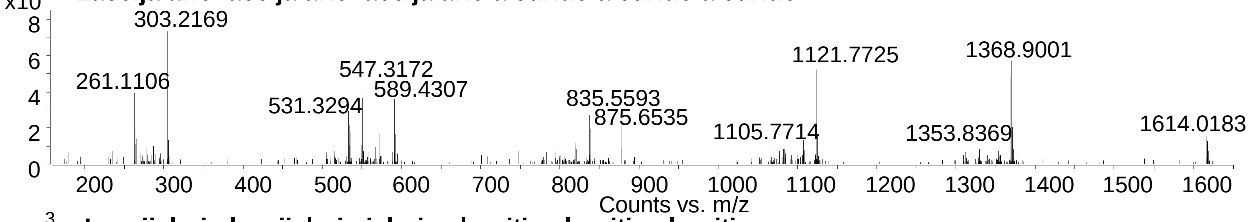

Laccijalaric-laccijalaric-jalaric-aleuritic-aleuritic-aleuritic

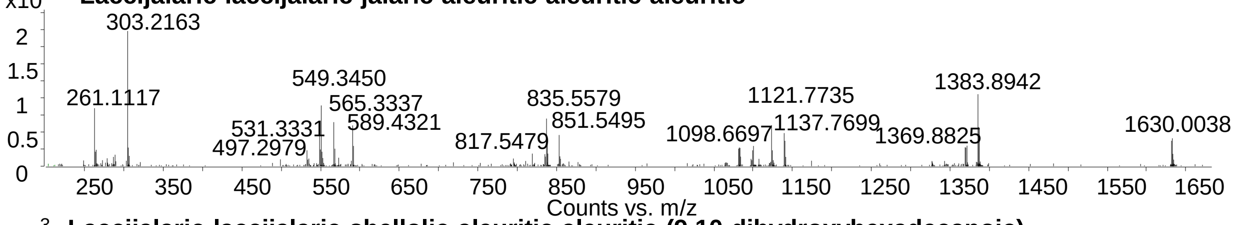

Laccijalaric-laccijalaric-shellolic-aleuritic-aleuritic-(9,10-dihydroxyhexadecanoic)

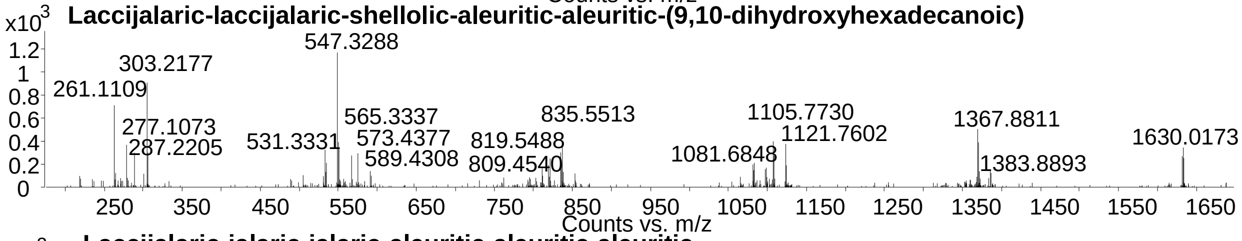

Laccijalaric-jalaric-jalaric-aleuritic-aleuritic-aleuritic

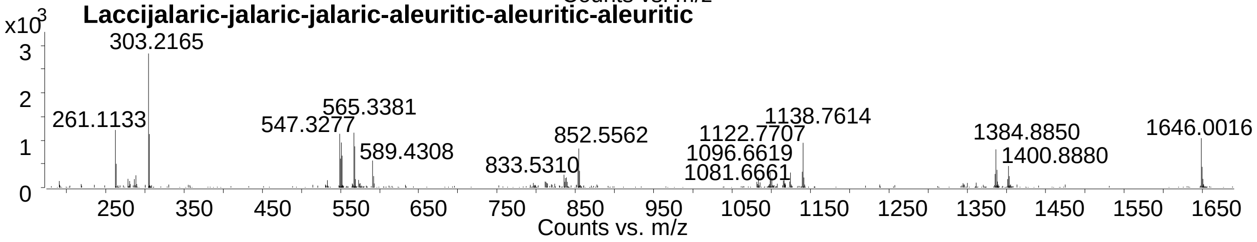

Laccijalaric-jalaric-shellolic-aleuritic-aleuritic-(9,10-dihydroxyhexadecanoic)

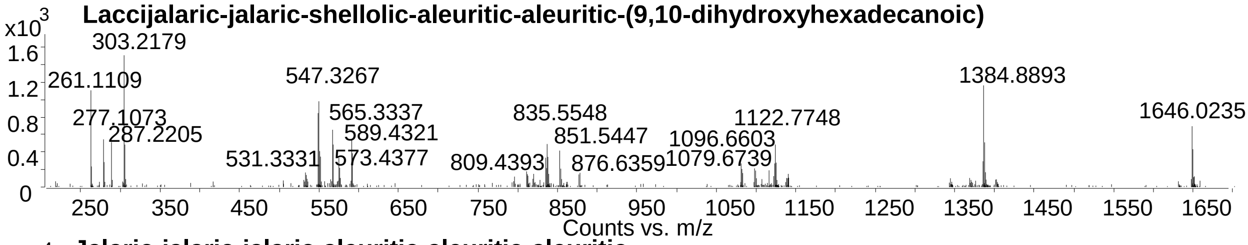

Jalaric-jalaric-jalaric-aleuritic-aleuritic-aleuritic

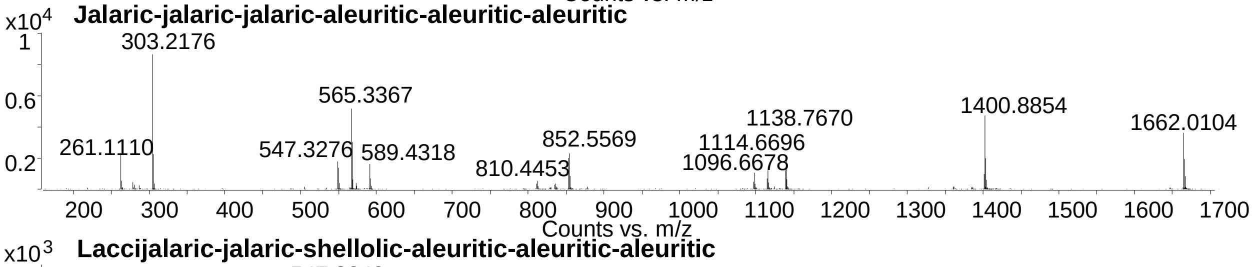

Laccijalaric-jalaric-shellolic-aleuritic-aleuritic-aleuritic

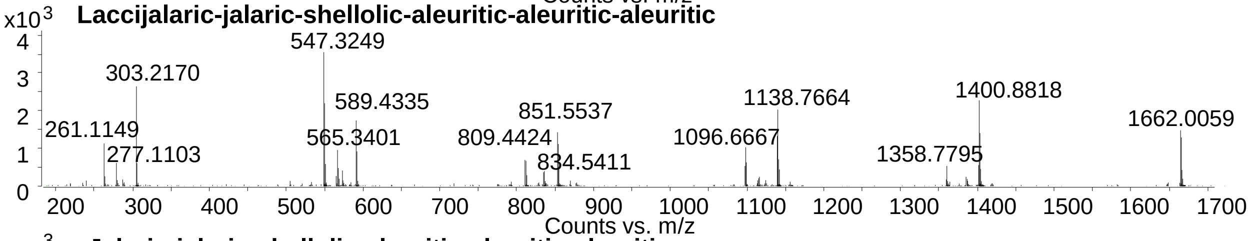

Jalaric-jalaric-shellolic-aleuritic-aleuritic-aleuritic

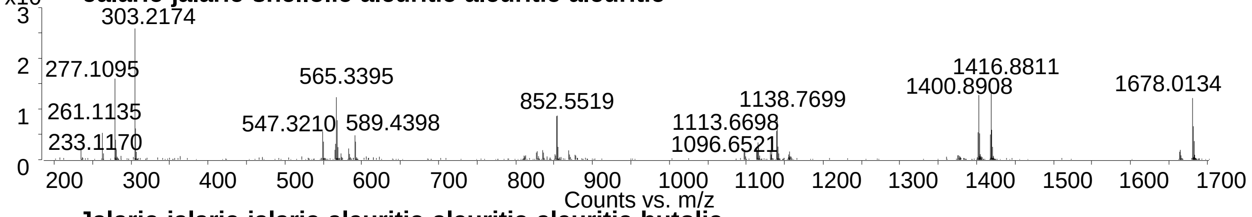

Jalaric-jalaric-jalaric-aleuritic-aleuritic-aleuritic-butolic

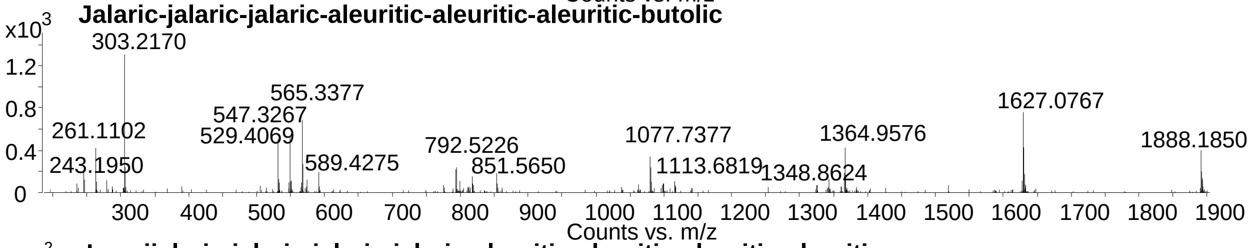

Laccijalaric-jalaric-jalaric-jalaric-aleuritic-aleuritic-aleuritic-aleuritic

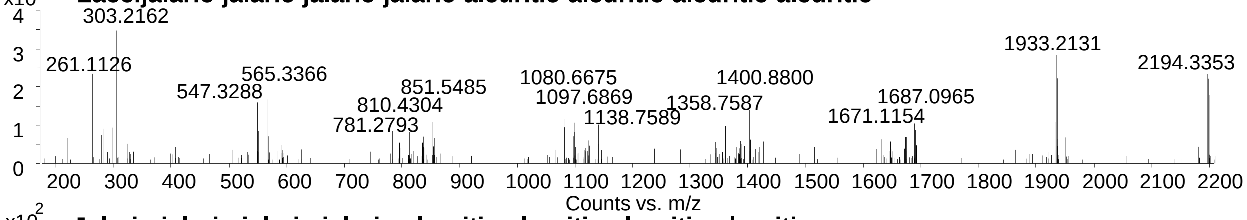

Jalaric-jalaric-jalaric-jalaric-aleuritic-aleuritic-aleuritic-aleuritic

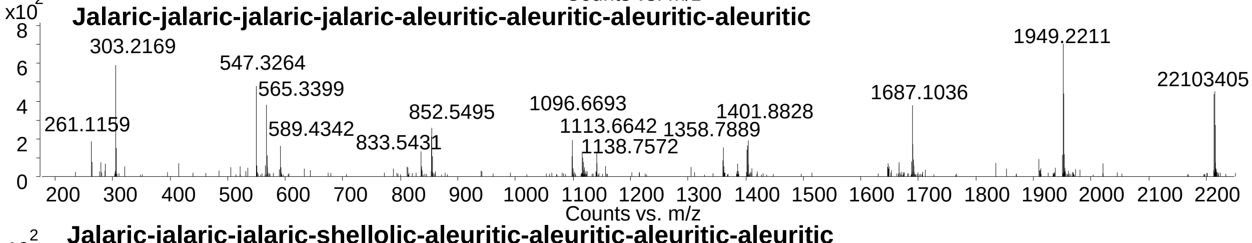

Jalaric-jalaric-jalaric-shellolic-aleuritic-aleuritic-aleuritic-aleuritic

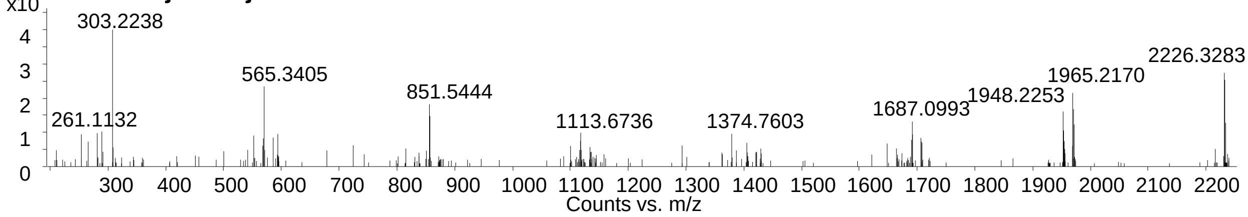

Supplement: Supplementary file 1 — Supplementary information [file 41598_2017_14907_MOESM1_ESM.pdf]
